# How do hospitals respond to feedback about blood transfusion practice? A multiple case study investigation

**DOI:** 10.1371/journal.pone.0206676

**Published:** 2018-11-01

**Authors:** Natalie J. Gould, Fabiana Lorencatto, Camilla During, Megan Rowley, Liz Glidewell, Rebecca Walwyn, Susan Michie, Robbie Foy, Simon J. Stanworth, Jeremy M. Grimshaw, Jill J. Francis

**Affiliations:** 1 School of Health Sciences, City University of London, London, United Kingdom; 2 Centre for Behaviour Change, University College London, London, United Kingdom; 3 Scottish National Blood Transfusion Service, Royal Infirmary of Edinburgh, Edinburgh, United Kingdom; 4 Leeds Institute of Health Sciences, University of Leeds, Leeds, United Kingdom; 5 Department of Health Sciences, University of York, York, United Kingdom; 6 Clinical Trials Research Unit, University of Leeds, Leeds, United Kingdom; 7 Transfusion Medicine, NHS Blood and Transplant, Oxford, United Kingdom; 8 Department of Haematology, Oxford University Hospitals NHS Foundation Trust, Oxford, United Kingdom; 9 Radcliffe Department of Medicine, University of Oxford, Oxford, United Kingdom; 10 Clinical Epidemiology Program, Ottawa Hospital Research Institute, Ottawa, Ontario, Canada; 11 Department of Medicine, University of Ottawa, Ottawa, Ontario, Canada; Macquarie University, AUSTRALIA

## Abstract

National clinical audits play key roles in improving care and driving system-wide change. However, effects of audit and feedback depend upon both reach (e.g. relevant staff receiving the feedback) and response (e.g. staff regulating their behaviour accordingly). This study aimed to investigate which hospital staff initially receive feedback and formulate a response, how feedback is disseminated within hospitals, and how responses are enacted (including barriers and enablers to enactment). Using a multiple case study approach, we purposively sampled four UK hospitals for variation in infrastructure and resources. We conducted semi-structured interviews with staff from transfusion-related roles and observed Hospital Transfusion Committee meetings. Interviews and analysis were based on the Theoretical Domains Framework of behaviour change. We coded interview transcripts into theoretical domains, then inductively identified themes within each domain to identify barriers and enablers. We also analysed data to identify which staff currently receive feedback and how dissemination is managed within the hospital. Members of the hospital’s transfusion team initially received feedback in all cases, and were primarily responsible for disseminating and responding, facilitated through the Hospital Transfusion Committee. At each hospital, key individuals involved in prescribing transfusions reported never having received feedback from a national audit. Whether audits were discussed and actions explicitly agreed in Committee meetings varied between hospitals. Key enablers of action across all cases included clear lines of responsibility and strategies to remind staff about recommendations. Barriers included difficulties disseminating to relevant staff and needing to amend feedback to make it appropriate for local use. Appropriate responses by hospital staff to feedback about blood transfusion practice depend upon supportive infrastructures and role clarity. Hospitals could benefit from support to disseminate feedback systematically, particularly to frontline staff involved in the behaviours being audited, and practical tools to support strategic decision-making (e.g. action-planning around local response to feedback).

## Introduction

Audit and feedback (A&F) is frequently used as a quality improvement strategy. It involves providing a summary of performance over a specific timeframe to healthcare organisations and staff [1]. Multiple national audit programmes in the UK, often commissioned by the Healthcare Quality Improvement Partnership, aim to identify and communicate performance differences between hospitals to stimulate practice change. However, A&F can generally only change practice if it is disseminated to relevant staff *and* if effective responses are initiated in light of the feedback received. Whilst there has been substantial research on optimising A&F by modifying attributes of the feedback (such as format and frequency) [1–3], there is relatively little research on processes following receipt of feedback and how these processes influence the likelihood of practice change. Better understanding of these factors is essential to optimise the effects of A&F.

**Fig 1:**
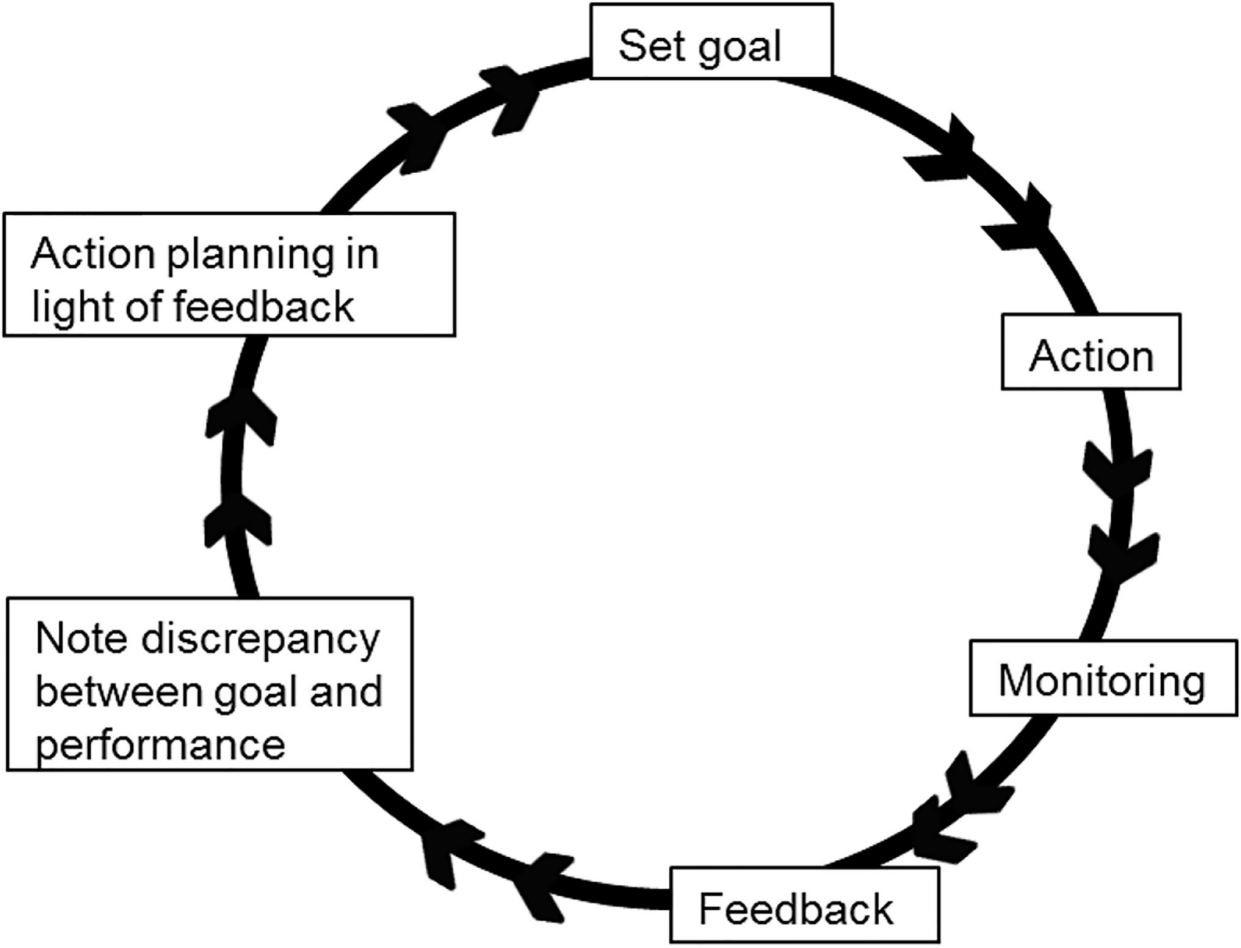
Control theory cycle [4].

Behavioural theories provide an additional dimension to explore *how* feedback might result in practice change, that is, to characterise the processes which occur *after* feedback has been provided that result in improvements to the quality of care provided by healthcare professionals. For example, Control Theory [4] (Fig 1) proposes that people regulate their behaviour through an iterative cycle. Feedback is a part of this cycle but the processes following receipt of feedback (e.g. noting discrepancies between current behaviour and an agreed standard); action planning) are vital to progressing around the cycle to the point of actual practice change. Hence, according to Control Theory, even the ‘perfect’ feedback document will not change practice unless it is followed by specific actions. However, like most actions taken in complex clinical contexts, actively managing a response to feedback requires staff and management to negotiate a range of potential barriers.

Some potential barriers to implementing recommendations from clinical audits have been identified in previous research, e.g. available resources, challenges relating to setting standards, motivational level of staff, and choice of audit topic [5, 6]. Further reported barriers and enablers include the relevance of quality indicators, credibility of the data, timeliness of the feedback, actionable feedback, transparency or credibility of the audit process and communication [7]. The effectiveness of A&F may also be influenced by including explicit goals and action plans in feedback documents and acknowledegements of good practice instead of primarily feeding back negative outcomes [8]. A&F interventions that target identified barriers to behaviour change are more likely to be effective than those which do not [9], but currently little is known about how healthcare organisations identify and address potential barriers, or how feedback is received.

Hence, understanding current practice is a first step in identifying how to support staff in the processes after feedback from an audit is received. In the United Kingdom, the National Health Service Blood & Transplant (NHSBT) National Comparative Audit (NCA) program undertakes audits of blood component utilisation to promote more appropriate use. Hospitals undertake local audits and provide their data to NHSBT, which provides feedback comparing hospital performance with national performance, and against audit standards. We undertook a multiple case study of how four hospitals received and acted upon NHSBT national audits to investigate who initially receives the feedback, how the feedback is disseminated within a hospital, who formulates a response to the feedback and how responses are enacted within a hospital (including barriers to, and enablers of, responding). This study was part of a wider research programme, AFFINITIE (audit and feedback interventions to increase evidence-based transfusion practice) [10, 11]. The protocol for the study reported here is published as part of the protocol for a larger portion of this programme [10].

## Methods

The Ethics Committee at City, University of London approved this study in October 2013 (Ref: Staff/13-14/09) and Research and Development approvals were obtained from each of the four participating trusts.

### Design

We used a multiple case study design involving semi-structured interviews and observations of Hospital Transfusion Committee (HTC) meetings at four hospital sites in England. We chose a multiple case study approach [12] to consider local contextual factors, exploring similarities and differences between hospitals, by comparing presence and frequency of themes identified in the interviews, comparing content of the themes, and comparing observational field notes from HTC meetings.

### Participants and sampling

#### Sampling of cases

Hospitals which routinely take part in the NHSBT NCAs of blood transfusion were identified in collaboration with the clinical leads on the research team. We used purposive sampling to identify four hospitals with diverse infrastructure (e.g. teaching or district general) and level of resources (number of transfusion practitioners). A member of the research team (NG) invited these hospitals through their respective Trust Research and Development offices.

#### Sampling of participants

We used purposive sampling to recruit participants involved in (i) prescribing blood components (ii) administering blood components to patients, and (iii) formulating and enacting change in response to feedback. To ensure that interviews were conducted with individuals responsible for a range of roles, we monitored sample diversity by recording participant characteristics (gender, clinical role, involvement in prescribing transfusions, length of experience, and previous involvement in A&F processes). We approached a local transfusion contact at each hospital to help identify potential interviewees and transfusion-related meetings to observe (e.g. HTC meeting). We emailed potential participants and invited them to participate. Those who agreed to participate gave written informed consent. We recruited additional participants opportunistically through recommendations from participants from individual interviews and observation of HTC meetings.

### Materials

We developed a semi-structured interview topic guide (S1 Appendix) and a structured observation sheet (S2 Appendix).

We used the Theoretical Domains Framework (TDF) [13] to develop the interview topic guide (Table 1). We chose to use the TDF as this framework has been applied across a range of clinical contexts to explore implementation problems, design interventions and investigate behaviour change processes (e.g., [14–17]), and TDF-based studies have been shown to elicit additional themes compared with interview studies without a theoretical basis [18, 19].

**Table 1:** Example topic guide questions for each TDF domain.

TDF domain	Description (adapted from [20])	Example question
**Knowledge**	An awareness of procedures, guidelines, evidence	What do you think about the audit standards? Do you find them credible?
**Skills**	Ability or competences	How much experience do you have in blood transfusion?
**Social/Professional role & identity**	The extent to which something is the individual’s job/responsibility, boundaries between professional groups	Is there someone who is responsible for receiving the feedback materials and feeding these back to the team?
**Beliefs about capabilities**	View about one’s confidence/ability to perform the target behaviour, self-efficacy, perceived behavioural control	Which changes would be easy and which more difficult?
**Beliefs about consequences**	View about the advantages and disadvantages, and the outcomes, of performing the target behaviour	What do you think are the downsides of changing blood transfusion practice in light of feedback?
**Motivation & goals**	The relative priority or importance of the target behaviour, intentions	Compared to other tasks that you have to do, where would you rank audit and feedback in terms of priority?
**Memory, attention & decision processes**	Level of attention needed to perform the behaviour, how decisions are made, memory to perform the behaviour	Do you remember which parts of the materials you looked at?
**Environmental context & resources**	Factors related to a person’s situation/setting in which the behaviour is performed (e.g. organisational, cultural, physical, financial)	Are there any constraints to the feedback process that we would need to address or work around if we were to make changes? (e.g. resources, time)?
**Social influences**	External influences/pressure from other people (e.g. other professions, colleagues, patients)	Did you discuss the feedback materials with any of your colleagues in the hospital?
**Emotion**	Affect (negative/positive), feelings	N/A*
**Behavioural regulation**	Ways of doing things in order to manage or achieve desired goals or standards, translating intention into action	Did you make any plans on how to change your practice or procedures to target these goals?
**Nature of the behaviours**	What has the person done in the past, are the (current) behaviours routine/automatic, how resistant are these behaviours to change?	Was there a specific meeting where you discussed the feedback?

*Following pilot interviews the question tapping the domain ‘emotion’ was removed

We first asked general questions to establish the extent of participants’ involvement in prescribing transfusions and their awareness of current A&F processes. We explored if and how feedback was discussed by staff, how responses to feedback were planned and methods used to support dissemination and responses to feedback (e.g. apps, posters and tools to facilitate ongoing monitoring of practice.

We piloted the topic guide with seven healthcare professionals with experience of A&F. The final topic guide (S1 Appendix) included 36 questions, with additional prompts, to elicit beliefs relating to 11 of the 12 domains. Following pilot interviews, we removed the question for the domain ‘emotion’ from the topic guide, as it was considered ambiguous by participants and did not elicit information distinct from responses to other questions. However, the domain ‘emotion’ remained in the coding scheme to ensure that any responses relevant to this domain were coded and not omitted.

We developed an observation sheet (S2 Appendix) to record field notes from HTC meetings, focusing on the format of the meeting, when/how A&F was discussed, purpose of the meeting (e.g. information provision, regulating of behaviour), group processes (e.g. leadership style, communication), signs of staff engagement (including body language), and group decision-making (whether and how actions were decided and agreed).

### Procedure

Two members of the research team (NG & FL) conducted face-to-face, one-to-one, semi-structured interviews lasting a maximum of one hour, at the hospital, at a time convenient for each participant. Both researchers observed one HTC meeting at each hospital, with the consent of the chair and attendees, and recorded field notes.

### Analysis

Interviews were audio-recorded, transcribed verbatim and anonymised. We classified participants into those who were part of the HTC, and those who represented the wider hospital, i.e. not members of the HTC. We analysed data from one hospital (Case 1) first, with data from subsequent hospitals (Cases 2, 3 and then 4) analysed using themes that had been identified in previous cases, whilst allowing other themes to emerge. If new themes were identified, we revisited earlier cases to check whether these themes were also present.

**Fig 2:**
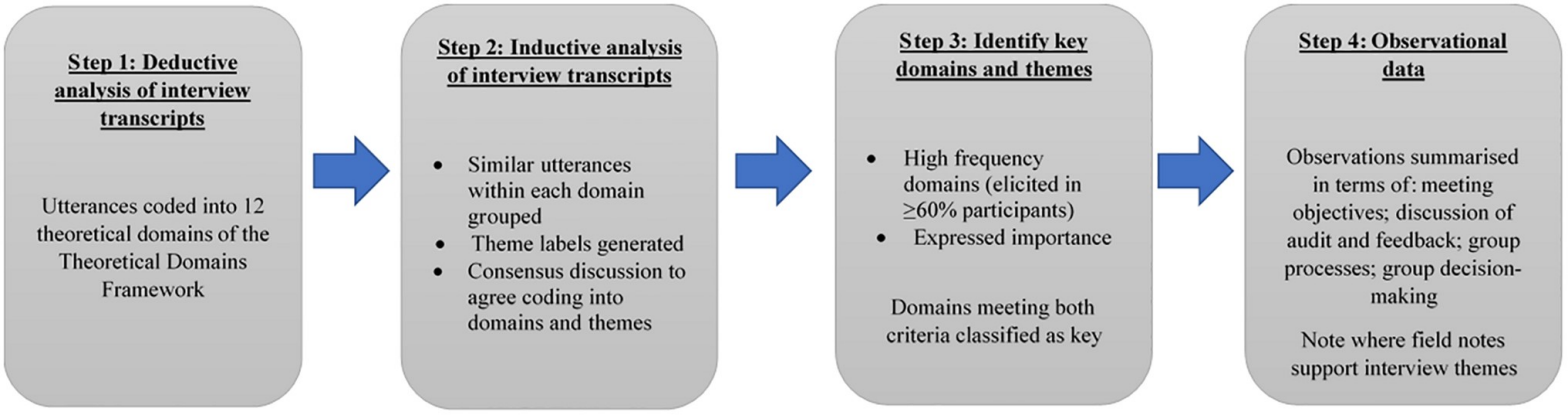
Stages of analysis for each case study.

We analysed data in four steps (Fig 2). First, we coded participant utterances to one or more of the TDF domains e.g. *“I rely on [Transfusion Practitioner] hugely to disseminate the findings among the nursing staff and on committee members to take it back to the medical teams”* [Case 1, P01] was coded into ‘social/professional role & identity’ and ‘social influences’.

Next, using an inductive thematic analysis approach, we grouped similar responses within each domain and generated a summary theme label. We combined themes identified in transcripts from both HTC and wider hospital participants and noted if HTC and wider hospital participants reported differing views. Where there were differing views about the same theme (i.e. some participants stated that something did occur/influence and others stated that it did not occur) we reflected this in the theme label with the addition of (not), or similar, for example ‘staff do (not) know about the NCA and audit process in general’. This indicates a range of views within that case, or across cases, for that theme. Two researchers (NG & CD) discussed and reached consensus on all themes, by examining each theme label alongside the utterances from the transcripts, discussing until agreement was reached that it was representative and correctly allocated to the domain(s). Theme names were reviewed by a third researcher (JF) before being finalised, with any disagreements resolved through discussion until full consensus was achieved.

We then identified key theoretical domains and themes. For each case, we reviewed domains against previously published criteria to assess their importance (e.g. [15, 21, 22]): (1) high frequency domains: domains that were mentioned by the majority (≥60%) participants in the hospital (for example, in a hospital with seven participants, at least five mentioned themes that were coded into that domain) and (2) expressed importance: where a participant spontaneously reported that a theme within the domain was important or problematic within their hospital. We classified domains as ‘key’ if they met both importance criteria.

Finally, we summarised observational field notes for each case to identify whether HTC meetings were used to initiate and/or manage responses to feedback from audits, how such meetings were conducted and which staff had attended. It was noted where observations provided examples of themes identified in the interview analysis.

## Results

### Characteristics of cases and participants

There were 25 interview participants; 16 were HTC members (Table 2).

**Table 2:** Case and participant characteristics.

Characteristic	Case 1	Case 2	Case 3	Case 4
Type of hospital	Acute General	District General	Teaching	Acute General
Size of hospital	750+ beds	500–600 beds	750+beds	600–700 beds
Location	SE England	Greater London	NW England	SW England
Number of transfusion practitioners	1	1 (no-one in this role for 2 years previously)	4	2
Total number of participants	7 (6 female, 1 male)	6 (3 female, 3 male)	7 (4 female, 3 male)	5 (2 female, 3 male)
Participants in HTC	Transfusion practitioner; laboratory manager; intensive care and A&E consultant	Transfusion practitioner; laboratory manager; haem-oncology consultant; **matron of surgery and urology**	Transfusion practitioner; patient blood management practitioner; audit facilitator; consultant anaesthetist; clinical transfusion lead/consultant	Transfusion practitioner; laboratory manager; consultant anaesthetist; consultant haematologist
Participants in wider hospital	**Acute care & intensive care consultant;** senior staff nurse (gastroenterology) **senior staff nurse (critical care); staff nurse (gastroenterology)**	**Advanced nurse practitioner;** lead clinical nurse specialist in haematology	Haematology registrar; **level 2 foundation doctor (gastroenterology)**	**Registrar**
Range of clinical experience of participants	4–30 years	4–25 years	2–27 years	1–34 years
Reported range of involvement in transfusion practice or following practice recommendations	Assessing signs and referring to doctor, policy & education, prescribing transfusions, influencing others’ prescribing of transfusions	Prescribing transfusions, influencing others’ prescribing of transfusions	Following practice recommendations for patient blood management, monitoring audits and implementation of action plans, Prescribing transfusions, influencing others’ prescribing of transfusions	Prescribing transfusions, influencing others’ prescribing of transfusions

Note. Roles in **bold** reported having had minimal involvement in previous NCA Audit & Feedback processes

### Who receives the feedback and formulates a response, and how are the audit results disseminated within a hospital?

**Fig 3:**
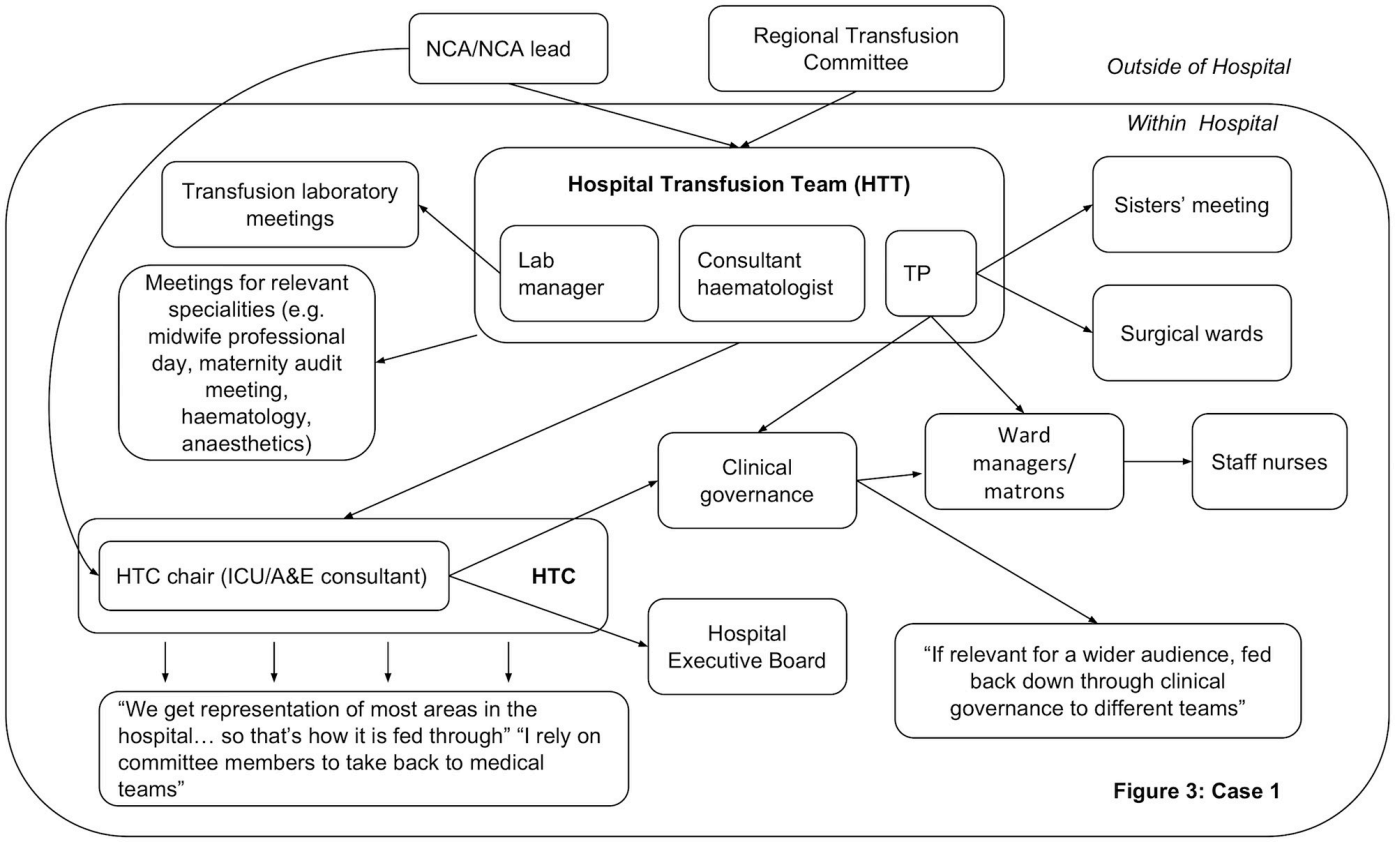
Reported dissemination pathway in Case 1 for national comparative audits (findings from interview data N = 7). A&E = Accident & Emergency; HTC = Hospital Transfusion Committee; ICU = Intensive Care Unit; NCA = National Comparative Audit; TP = Transfusion Practitioner.

**Fig 4:**
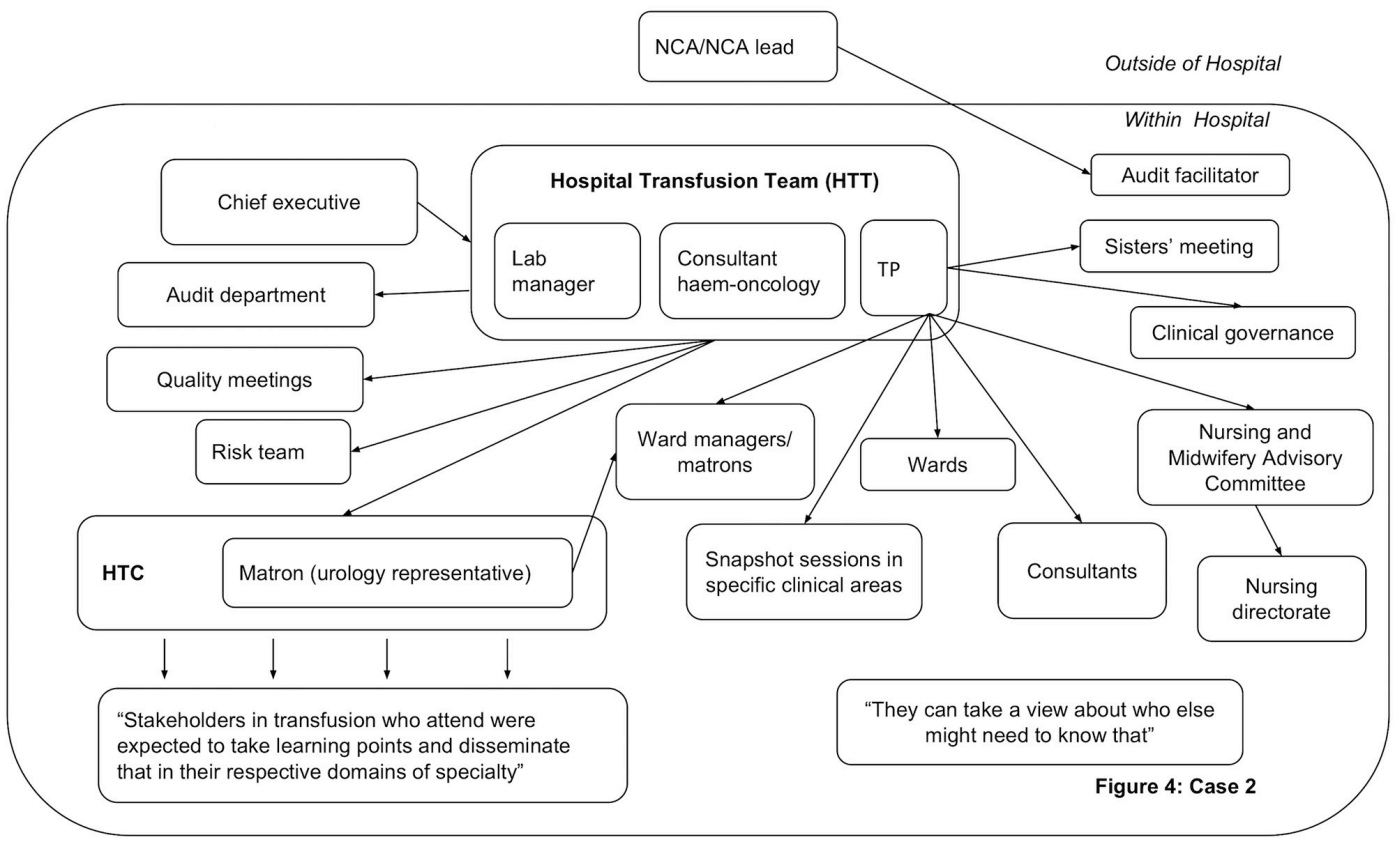
Reported dissemination pathway Case 2 for national comparative audits (findings from interview data N = 6). HTC = Hospital Transfusion Committee; NCA = National Comparative Audit; TP = Transfusion Practitioner.

**Fig 5:**
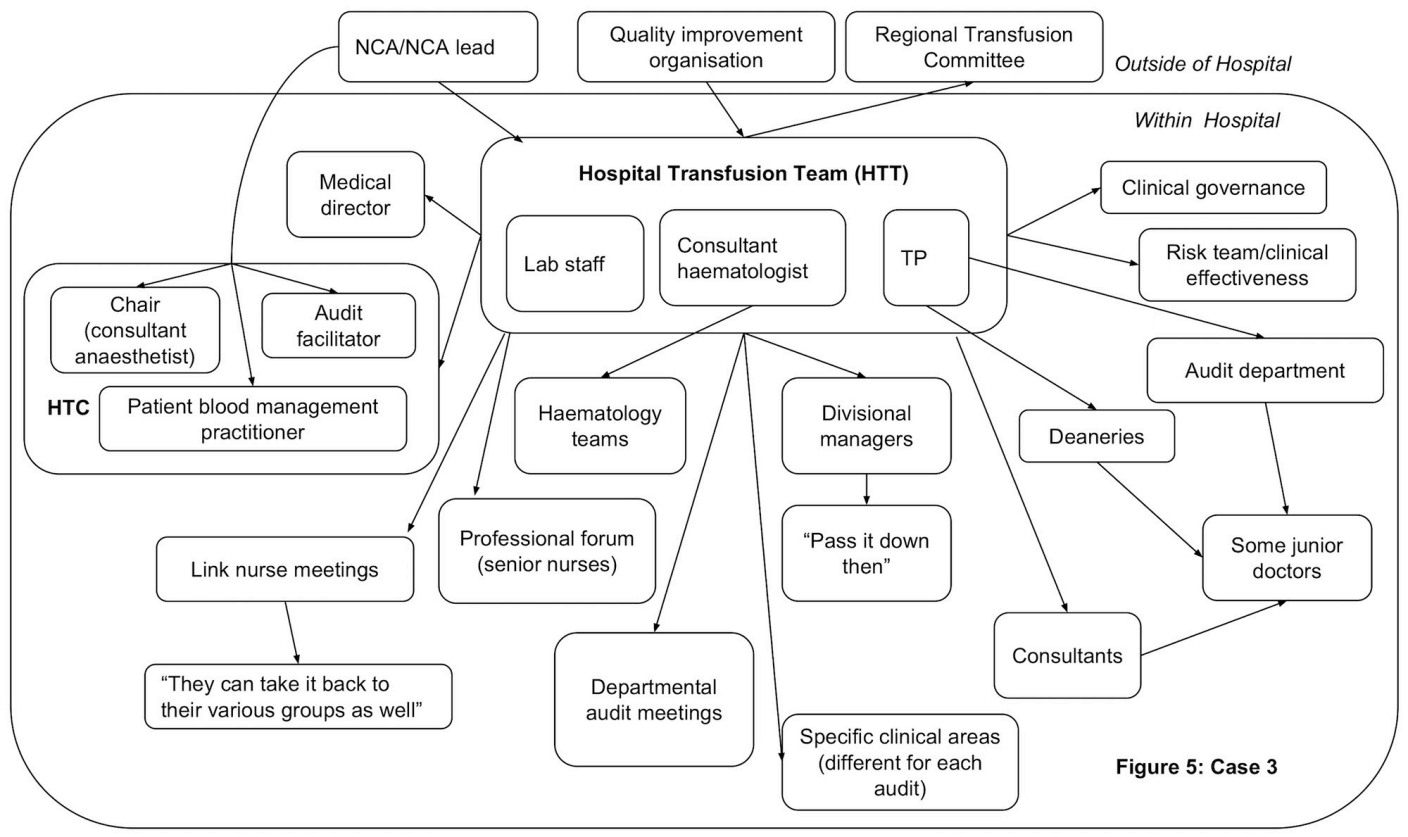
Reported dissemination pathway Case 3 for national comparative audits (findings from interview data N = 7. HTC = Hospital Transfusion Committee; NCA = National Comparative Audit; TP = Transfusion Practitioner.

**Fig 6:**
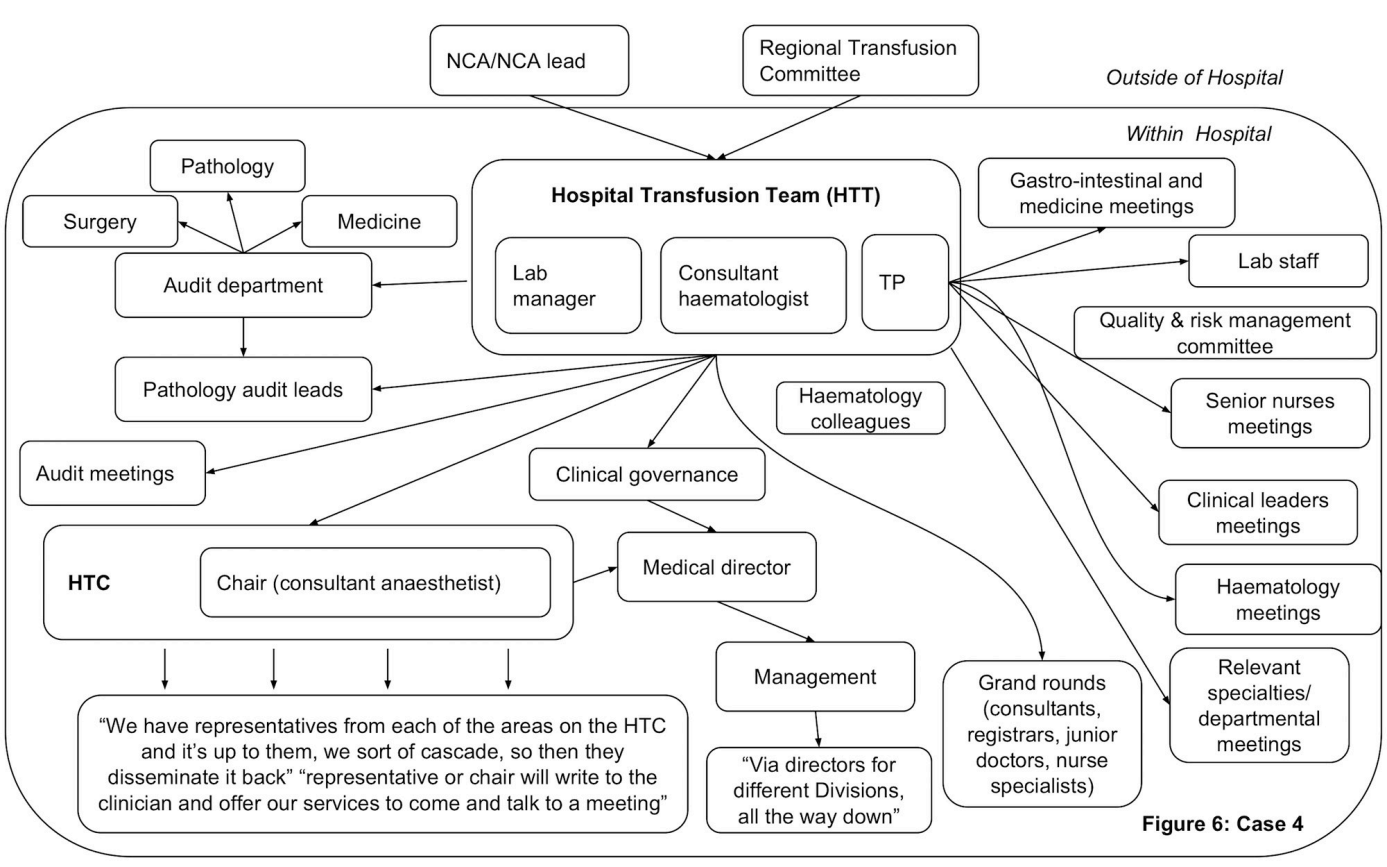
Reported dissemination pathway Case 4 for national comparative audits (findings from interview data N = 5. HTC = Hospital Transfusion Committee; NCA = National Comparative Audit; TP = Transfusion Practitioner.

In all hospitals, dissemination activity was reported demonstrating that feedback was circulated beyond the initial recipients. However, dissemination did not always reach the individuals prescribing blood components. Figs 3–6 present the reported dissemination pathway for each participating hospital. Across all cases, members of the Hospital Transfusion Team (typically, a transfusion practitioner(s), consultant haematologist, and transfusion laboratory manager) were the initial recipients of the feedback from the NCA. In some cases, the NCA office also disseminated feedback directly to others in the hospital, notably, the audit facilitator (Cases 2 and 3, Figs 4 & 5), and the HTC chair (Cases 1 and 3, Figs 3 and 5). Aside from the audit facilitator in Case 2 these other individuals were part of the HTC.

The HTC was an established infrastructure to facilitate dissemination of feedback about blood transfusion practice and formulation of a local response to the feedback. The HTC was made up of the Hospital Transfusion Team and representatives from various specialities across the hospital (e.g. anaesthesia, obstetrics, orthopaedics), along with roles such as audit facilitators and patient blood management practitioners. Members of the Hospital Transfusion Team disseminated the feedback documents to the HTC members. In addition, the Hospital Transfusion Team disseminated the feedback to selected staff in the wider hospital (e.g. through relevant specialty meetings; through clinical governance procedures), with an assumption that representatives on the HTC would disseminate within their specialties. In all hospitals, attempts were reported to disseminate feedback to senior management such as the chief executive, the executive board or the medical director.

However, within each hospital, some interviewees reported not receiving feedback from a national audit of blood transfusion, despite being involved in prescribing or administering blood components to patients. For example, a consultant working in acute care and intensive care said, *“The fact that I haven’t really seen it [feedback] means there must be some problem…I really am not sure I’ve ever had an email about it”*. Of the nine participants interviewed from outside of the HTC, six reported that they had not received feedback in previous national comparative A&F cycles (Table 2), indicating a breakdown in the dissemination pathway to front-line staff involved in prescribing blood components.

### How are responses enacted within hospitals and what are the barriers and enablers?

We present the themes that were present in high frequency (≥60% participant interviews) across all cases first, with summaries of HTC meetings in terms of what was consistently observed. For each case, we then present where additional high frequency themes were identified and differences between observations of HTC meetings. Finally, we identify high key theoretical domains, and any differences between members of the HTC and staff from the wider hospital.

#### High frequency themes across all cases

The key themes that were high frequency across interviews from all cases are displayed in Table 3 and example quotes can be found in S1 Table.

**Table 3:** High frequency themes (present in ≥60% participants) in interviews from all cases.

Theme	Frequency of participants					Theoretical Domain
	Case 1 (n = 7)	Case 2 (n = 6)	Case 3 (n = 7)	Case 4 (n = 5)	Total (n = 25)	
Feedback is (not) shared and discussed with the relevant staff ^a^	7* (2+/5 =)	6* (3+/1-/2 =)	7 (2+/5 =)	5* (2+/3 =)	25 (9+/1-/15 =)	Social influences
Feedback should come from someone whom staff know or respect, to influence change	5	4	5*	4	18	
I (do not) have influence over practice change ^a^	7 (3+/1-/3 =)	6 (+)	7 (6+/1-)	4 (+)	24 (19+/2-/3 =)	
Comparing our performance against national performance is (not) useful for identifying areas for improvement ^a^	6 (3-/3 =)	6* (1+/2-/3 =)	7* (4+/1-/2 =)	4* (3+/1 =)	23 (8+/6-/9 =)	
We have to amend the feedback to make it relevant to our hospital	5	4	6	4*	19	Behavioural regulation
We try to monitor practice by re-auditing, re-feeding back and following up	6*	5	7*	4	22	
We (do not) set goals or make action plans as a team ^a^	6* (3+/1-/2 =)	6 (4+/2 =)	6 (3+/1-/2 =)	4* (+)	22 (14+/2-/6 =)	
Support materials could be useful for some staff	6	6	7	5	24	
We need or use strategies to remind staff of actions and recommendations	6*	4*	6	4	20	
It is clear who is responsible for audit and feedback	6	6	6	4	22	Social/professional role & identity
Staff (do not) know about NCA audits ^a^	7 (4+/3-)	5 (2+/3-)	7 (6+/1-)	5 (4+/1-)	24 (16+/8-)	Knowledge
Other demands take priority over responding to audit and feedback	6	6	7*	4	23	Motivation & goals
We require sufficient staff to conduct audits and/or respond to feedback	6	5	6*	5*	22	Environmental context & resources
Audit and feedback does (not) influence practice change ^a^	6 (5+/1-)	6 (4+/2-)	7 (4+/3 =)	4 (2+/1-/1 =)	23 (15+/4-/4 =)	Beliefs about consequences
I (do not) remember feedback materials ^a^	5 (3-/2 =)	4 (3+/1-)	5 (3+/2 =)	4 (2+/2 =)	18 (8+/4-/6 =)	Memory, attention & decision processes
I notice only information that is new, ‘leaps out’ as different or is clinically relevant to me	6	6	7	3	22	

^a^ (‘not’ or ‘do not’) indicates participants expressed differing views in the same theme: positive (+), negative (-), both positive and negative (=);

* denotes expressed importance by one or more participants

In terms of the domain, social influences, across all cases participants reported that feedback was not always shared and discussed with relevant staff (as also highlighted above in dissemination pathways) and that national feedback was not always considered a useful comparator for identifying areas for improvement as; “*comparing yourself with like-for-like trusts is more valuable than comparing yourself with a trust that’s got a totally different sort of clinical activity to you*” [Case 2 P02]. To enable response to feedback it was suggested that feedback should come from someone whom others knew or respected.

Participants reported acting on feedback by goal-setting and creating action plans following feedback, although in all cases except Case 4 some participants stated that this did not always happen (mostly those from the wider hospital rather than the HTC). Attempts to monitor practice through re-auditing and feeding back were also reported; “*if in the audit when there is recommendations and we have sat and made plans to implement them*, *then I will say we re-audit six months later… to see if it is actually working or not*” [Case 3 P02]. Support materials were considered potentially helpful for some staff, along with strategies to remind staff of recommendations, such as timers, stickers, posters or pop-ups on the computer system. A perceived barrier to enacting the feedback was the need to amend feedback to make it locally relevant, for example “*there’s a risk that the recommendations can be quite broad-based and not specific for your own hospital*, *which is why we try and translate the recommendations… into more locally do-able actions”* [Case 4 P01].

Clear lines of responsibility for A&F within the hospital were identified as enablers by participants, including conducting the audit, disseminating the feedback and results, discussing the feedback, responding to the feedback and enacting any associated action plans, for example “*it [feedback] would be fed back to me saying that this is what we are not doing well and this is what we need to put in place*, *so then I would have to make sure that that was executed by our SPOT and in our transfusion laboratory and the other colleagues”* [Case 2 P04] although knowledge of NCA audits was varied. Participants also reported barriers including competing priorities, not having sufficient staff for conducting audits and responding to feedback, not always believing A&F changed practice and not always remembering the feedback.

HTC meetings were held every three to four months at each hospital. A range of staff attended, representing a number of disciplines (Table 4), and all meetings followed a structured agenda and discussed patient safety and blood wastage. Meetings differed in leadership, discussion of A&F, and setting of action points. Differences are summarised within each case below.

**Table 4:** Staff roles present at the Hospital Transfusion Committee meetings.

	Case 1	Case 2	Case 3	Case 4
**Chair**	Consultant anaesthetist	✓	✓	✓
**Other roles**	Transfusion practitioner	✓	✓	✓
	Lab manager	✓	✓	x
	Matron	✓	x	x
	Consultant, haematologist	x	✓	✓
	Consultant, obstetrics	x	✓	x
	Consultant, renal	x	x	x
	Consultant, orthopaedic	x	x	x
	Regional transfusion practitioner	x	x	x
		Patient blood management practitioner	✓	x
		Audit facilitator or practitioner	✓	x
		Clinical nurse specialist	x	x
		Ward sister	x	x
		Nurse, paed-oncology	x	x
		Junior doctor	✓	x
		External rep from manufacturer	x	x
			Consultant, anaesthetist (not the Chair)	✓
			Advanced nurse practitioner	x
				Senior staff nurse
				Transfusion co-ordinator
				Consultant, Emergency

#### Differences in high frequency themes between cases

Table 5 shows where high frequency interview themes differed across the four cases. As shown, the majority of themes were present in more than one case, but not always with high frequency. Each case is discussed separately below, with interview findings presented first.

**Table 5:** Differences in high frequency, or presence of, themes across cases.

Theme	Case 1	Case 2	Case 3	Case 4	Theoretical Domain
Staff use inductions, training sessions and study days to influence practice	✓	✓*	✓✓	✓	Social influences
External sources, such as patients, regional meetings and authorities, influence response to audit and feedback	---	✓✓	✓✓	✓✓	
I do (not) have support from my colleagues to make changes following feedback	---	✓ (2+/2 =)	✓✓ (2+/2-/2 =)	✓✓ (1-/3 =)	
The time between data collection and feedback is (not) too long	✓* (3+)	✓ (1+/2-)	✓✓* (6+)	✓✓* (3+/1-)	Behavioural regulation
We analyse our data and feed back or act immediately rather than wait for the national feedback	✓*	---	✓	✓✓	
Key individuals are (not) at meetings to discuss and disseminate feedback to their specialities	✓ (2+/2 =)	✓ (1+/2 =)	✓✓ (3+/2-)	✓✓* (2-/1 =)	Social/Professional role & identity
Some clinical disciplines are more receptive to change than others	✓	✓	✓	✓✓*	
Having specialist nurses or champions has raised the visibility and dissemination of feedback	---	✓✓*	✓	✓	
The nature of transfusion itself can make it difficult to follow recommendations	✓✓	✓	✓	✓	Nature of the behaviours
Established practices make it difficult to implement change	✓*	✓*	✓✓*	✓*	
The feedback materials are too long	✓*	✓	✓✓	✓	
Staff (do not) have knowledge of blood transfusion	✓✓* (3+/4-)	✓✓* (2+/2-)	✓ (3+/1 =)	✓✓ (2+/1 =)	Knowledge
Feedback is (not) clinically appropriate, or valid, or credible	✓✓ (3+/2-)	✓ (3+)	✓✓ (6+)	✓✓ (3+)	Motivation & goals
Staff are (not) enthusiastic about audit and feedback	✓ (1+/2-/1 =)	✓✓ (1+/2-/1 =)	✓✓ (6 =)	✓✓ (2+/2 =)	
Standards are (not) up to date, or appropriate, or credible	✓ (1+/2 =)	✓ (3+)	✓✓ (3+/2 =)	✓✓ (2+/1 =)	
I experience ‘audit fatigue’	---	✓	✓✓	✓✓	
Feedback highlights that change is needed to enhance patient safety and outcomes	✓	✓	✓✓	✓✓	Beliefs about consequences
Audit and feedback does (not) reduce costs	✓ (1+)	✓ (1+)	✓ (2+/1 =)	✓✓ (3+/1 =)	
Staff (do not) remember recommendations and action plans	✓✓ (1+/4-/1 =)	✓ (1+)	✓✓ (2+/3-/1 =)	✓✓ (3-)	Memory, attention & decision processes

Note: ✓ denotes presence of a theme; ✓✓ denotes presence of theme in high frequency of participants; * denotes expressed importance;---denotes absence of a theme; (not) indicates a theme where participants expressed differing views along the same theme or ‘dimension’;–indicates number of participants who expressed a view consistent with the word in brackets e.g. (not), + indicates number of participants who expressed a view consistent with the theme; = indicates number of participants who expressed views on both the negative and positive side of the theme.

In Case 1, in addition to the findings presented across all cases, a high frequency of participants reported four perceived barriers to responding to feedback, within nature of the behaviours, motivation & goals, knowledge and memory, attention & decision processes. The nature of transfusion itself was reported as a barrier to following recommendations, for example it is sometimes more cost-effective to give patients a transfusion rather than alternative therapies. Feedback was sometimes perceived not to be clinically appropriate or valid, staff had varied knowledge of blood transfusion practice, and recommendations or action plans were not remembered.

The HTC meeting in Case 1 was led by the committee chair (a consultant anaesthetist) and the transfusion practitioner, with evidence of good rapport and engagement by members. Audit findings were discussed, in response to a recent NCA, and there was discussion around key transfusion events that may have influenced their performance (supporting the theme that the nature of transfusion itself can make it difficult to follow recommendations). Further discussion focused on goal setting and action planning in relation to feedback, re-monitoring performance, and comparing performance with other hospitals, which corresponds to the interview findings (some of the themes presented in Table 3). However, action points were not summarised or explicitly agreed by the group, but these were to be circulated following the meeting.

Participants in Case 2 reported that the ‘weight’ of national-level feedback external to their hospital influenced responses to A&F, for example, “*one of the advantages of transfusion practice is because it’s quite…regulated*, *people tend to see if something’s coming from NHS BT*, *they… think*, *“Well*, *we don’t have a choice*, *we have to implement this”*, *so yeah*, *it helps if something carries national weight*” [P03]. The appointment of specialist nurses or champions in transfusion was perceived to have raised the visibility and dissemination of feedback, a belief that was particularly salient in this hospital due to the previous absence of such a role, for example the transfusion practitioner stated; “*Change in practice is encouraged by my training and me being very visible*. *And because they hadn’t had that in the past five years*, *they see me as that leader and making sure* … *especially the shop floor staff*, *the front-line staff see me as having*, *since I have been here*, *making this improvement*”. Like Case 1, participants reported that staff had varied knowledge about blood transfusion practice. Finally, participants also reported that staff were not always enthusiastic about audit and feedback which could be a barrier to responding to feedback.

The HTC meeting in Case 2 involved group discussion rather than being led by the chair (a consultant anaesthetist). There was little discussion about audit, this being mentioned only at the end in relation to waiting for feedback from the recent NCA. Time-keeping was problematic and resulted in the last part of the meeting being rushed. Again, action points were not explicitly summarised or agreed, although there was some discussion about what to do next in relation to policy change.

In Case 3, a further nine high frequency themes were identified, although all had been mentioned by participants in previous cases. Participants reported using staff inductions or study days to influence practice, for example ‘*we’ll sometimes put a PowerPoint in the mandatory training…highlighting the feedback of audit*, *and that impacts on a wider range of staff*’ [P04], although sometimes staff did not feel supported by colleagues in making changes.

Several barriers to making changes following feedback were identified by Case 3 participants: the timing of feedback compared to when data were collected for the audit: “*by that time you may already be onto the next audit and then don’t have time to implement the recommendations from the previous audit*” [P01]; key individuals were not always at meetings to discuss and disseminate feedback; established practices were resistant to change: “*‘we’ve always done it this way*, *why should I change*?*’… pushing against culture… perhaps the hardest thing to change*” [P02]; length of feedback reports; perceived credibility of audit standards; and experiencing ‘audit fatigue’: “*I think there’s a danger that there can be too many audits*, *so that people get a bit audit fatigued*. *Particularly as we’re expected to do local and regional audits as well as national audits” [P01]*. Feedback was considered to be clinically appropriate and credible, and perceived to highlight how patient safety and outcomes could be enhanced.

The HTC meeting in Case 3 was led by the consultant haematologist rather than the committee chair (a consultant anaesthetist). The chair, consultant haematologist and transfusion practitioner were the main contributors to the discussion and a formal communication style was used in comparison to other cases. There was variable engagement across the meeting (supporting the interview theme of varied enthusiasm of staff), and a number of absences (recorded as apologies), reflecting that key representatives were not always present. Like Case 1, audit was discussed, focusing on local and national audits, and re-monitoring performance. Similar to previous cases, action points were not set or agreed in the meeting but were to be discussed in a subsequent meeting of the Hospital Transfusion Team.

In Case 4, a high frequency of participants reported analysing their own data and feeding back to colleagues before receiving the national feedback; perceiving some clinical disciplines as more receptive to change than others (for example, “*nursing staff are much more receptive to advice and change than doctors*” [P02], and “*the hardest ones to influence are normally surgery*. *The easiest ones are medicine*” [P01]); and having mixed views about whether A&F reduced costs. Like Case 3, a high frequency of participants perceived the feedback as clinically appropriate and credible.

The HTC meeting in Case 4 was led by the committee chair (a consultant anaesthetist) and consisted mainly of group discussion. Audit was discussed in relation to local and national audits. Unlike the three previous cases, clear, explicit action points were set and agreed in the meeting and decisions were made collaboratively by the group. There were a number of apologies and there was some discussion focused around how to engage or change practice in groups that were not present at the meeting (supporting high frequency themes of key representatives not attending, varied enthusiasm of staff, and some disciplines being more receptive than others). In addition, the committee suggested that feedback or attempts to engage other staff would be better received if it came from the chair because he was known to the staff groups, supporting the suggested enabler of feedback coming from someone that staff know or respect.

#### Differences in views between HTC members and wider hospital staff

Across all four cases there was little divergence of views between staff from the HTC and those in the wider hospital, and most themes were identified in both staff groups. There were only three areas where staff groups differed in their views. First, in general, HTC members reported knowing about the NCA and audit processes, whereas those from the wider hospital reported a lack of knowledge. Linked to this, most HTC members remembered feedback materials from an audit; those in the wider hospital generally did not remember. Finally, HTC members generally stated that A&F was a high priority whereas those in the wider hospital stated that clinical responsibilities take priority over A&F.

#### Key theoretical domains

Applying the criteria described in the methods, potential targets for intervention are centred around key barriers to, and enablers of, feedback within nine theoretical domains; professional responsibility for the response (all cases), influences of others (all cases), how behaviour is regulated (all cases), the nature of established practice (all cases), knowledge of the audit and the feedback (Cases 1 and 2), motivation and competing priorities (Cases 1 and 3), available resources (Cases 3 and 4), beliefs about the consequences of responding (Case 4) and recollection of feedback (Case 4).

## Discussion

National clinical audits are considerable undertakings; their successful conduct depends upon high levels of national and local professional commitment and resources. However, the success of their impact may vary markedly at local levels. Within four UK hospitals, we found similar processes for receiving feedback and developing the initial action plan, but variations in how feedback was disseminated or enacted, particularly in ensuring that front-line staff were aware of both the feedback and the proposed action plan. Our findings also suggest that there are tangible and realistic ways of enhancing the local, and hence wider, impact of national audits.

Across all four hospitals the Hospital Transfusion Team were the initial recipients of the feedback from the NCA and were then responsible for deciding how feedback was disseminated within the hospital, and to whom, and for co-ordinating the response. Staff outside of the Hospital Transfusion Team were aware of these lines of responsibility, and the creation of specific transfusion specialist roles was perceived to have raised the visibility, and dissemination, of feedback. A lot of dissemination activity was reported, often enabled through the infrastructure of the HTC, but subsequent pathways varied. Crucially, in each case, there were key staff from the wider hospital (e.g. consultants working in intensive care) who had never received feedback. Therefore, if the feedback was not reaching the staff whose behaviour is being audited, it would not lead to a change in behaviour, and therefore have little impact on patient safety or outcomes. These findings indicate opportunity for improving the reach of feedback to relevant staff, particularly through formalising the pathway of dissemination from the HTC to the wider hospital staff who are not part of the committee, perhaps by identifying specific contacts within key target groups. In addition, encouraging feedback to be disseminated by someone whom staff know or respect may help to influence effective responses to the feedback, as identified as an enabler in this study, and as recommended in previous literature [1–3].

The HTC meeting brings together representatives from key areas of the hospital to disseminate feedback and facilitate an active response through strategic level discussions about prioritising goal-setting and the ongoing monitoring of practice, activity that has a good fit with Control Theory [4]. However, key representatives were not always present at the HTC meetings, audit and feedback was not always discussed, nor explicit actions set and agreed. Therefore, response to feedback may not be enacted to full potential, likely influencing the effectiveness of the reach of the feedback and the quality and scale of plans to change practice (i.e. the processes associated with Control Theory [4]), suggesting meetings or processes could be optimised.

In addition, a number of barriers to responding to feedback were identified indicating room for efficiency gains and greater impact of the feedback, such as a lack of knowledge about the NCA or blood transfusion practice, lack of influence over change, lack of support to make changes, and the need to amend feedback for local use. This suggests that support could be provided to facilitate practice change, and reports could be re-structured to increase efficiency at the hospital level by reducing the time and effort staff reported spending on analysing their own hospital’s data and amending feedback for local use.

Some of the barriers in this study were related to attributes of the feedback (for example, national comparators were not always considered the most useful, feedback documents too long and too long a delay between data collection and feedback). There are evidence-based recommendations about the attributes of feedback documents that make them more effective for improving clinical practice; feedback practice should observe these recommendations [1–3]. Also, some barriers and enablers identified in this study converge with findings in previous A&F research from other clinical areas, for example problems with data credibility [5, 7] and motivational level of staff [6], suggesting that the findings may inform A&F interventions more generally and not just within the context of blood transfusion.

Across the four cases the themes identified were mainly the same, with some differences in whether they were expressed by a high frequency of participants, while only a few themes were hospital specific. This suggests that broad level interventions could be designed to support staff in their response to feedback, whilst allowing for tailoring to ensure relevance to their own local context. Key theoretical domains were identified which provide evidence to support theoretically-informed approached to enhancing feedback processes. In line with Control Theory [4], we found three key behaviours that could be targeted to enhance A&F processes; disseminating the feedback to relevant colleagues, planning and implementing changes (tailored to local context), and monitoring ongoing practice.

### Strengths and limitations

This study was embedded within a national audit programme, which is a cost-effective way to conduct highly contextualised research [23]. Although it is possible that the presence of observers may have influenced the ways in which committee meetings were conducted, the multi-method approach adopted in this study enabled some verification between self-report data and observational data. Although the sample size was small (four hospitals, 25 interviewees), cases were purposively sampled to ensure diversity, and no new themes emerged in Case 4, indicating that thematic saturation had been reached. The approach to analysis in this study has attempted to use both quantitative (frequency) and qualitative (expressed importance) approaches to comparing interview findings between cases and identifying key theoretical domains [21]. Although there are varying professional roles in each case which could influence whether a theme is identified as high frequency, all roles were involved in key transfusion or audit-related behaviours and there were few differences in the views of those from the HTC or from the wider hospital suggesting this was an appropriate approach. The importance criteria used to identify key TDF domains were informed by previous studies [15, 21, 22] and aimed to focus on a small number of key barriers and enablers rather than the whole range that were elicited in the interviews. Yet, there is little consensus in the field on thresholds for determining key domains or themes. Different methods for identifying importance could result in different domains being prioritised [21]. Hence, future research could explore this as a methodological question.

## Conclusions

This study explored hospital-based responses to audit and feedback, identifying potential for gains in the efficiency and effectiveness of audit and feedback. Consistent findings across four diverse cases suggested that, in the context of blood transfusion practice, infrastructure and role clarity are features of the hospitals that facilitate appropriate responses to feedback but opportunities for improvement were also identified, particularly in ensuring that feedback reaches the relevant frontline staff involved in the target behaviours. Hospital Transfusion Teams could benefit from: support for more systematic dissemination of feedback documents throughout the hospital and practical tools to support strategic decision making (e.g., for action planning and identifying locally-agreed targets for improving practice). While this study investigated responses to feedback in the context of blood transfusion practice, the findings suggest strategies that may enhance the impact of audit and feedback across a wide range of clinical areas.

## Supporting information

S1 Appendix Semi-structured interview topic guide. (DOCX)

S2 Appendix Observation sheet for field notes. (DOCX)

S1 Table Example quotes for each high frequency theme at each case. (DOCX)

## References

[1] Ivers NM, Sales A, Colquhoun H, Michie S, Foy R, Francis JJ, et al No more ‘business as usual’with audit and feedback interventions: towards an agenda for a reinvigorated intervention. Implementation Science. 2014;9(1): 1. 24438584 10.1186/1748-5908-9-14 PMC3896824

[2] Ivers N, Jamtvedt G, Flottorp S, Young JM, Odgaard-Jensen J, French SD, et al Audit and feedback: effects on professional practice and healthcare outcomes (Review). The Cochrane Library. 2012;7. 10.1002/14651858.CD000259.pub3 PMC11338587 22696318

[3] Brehaut JC, Colquhoun HL, Eva KW, Carroll K, Sales A, Michie S, et al Practice Feedback Interventions: 15 Suggestions for Optimizing Effectiveness. Annals of internal medicine. 2016;164(6): 435–41. 10.7326/M15-2248 26903136

[4] Carver CS, Scheier MF. On the Self-Regulation of Behaviour. Cambridge: Cambridge University Press; 1998.

[5] D’Lima DM, Moore J, Bottle A, Brett SJ, Arnold GM, Benn J. Developing effective feedback on quality of anaesthetic care: what are its most valuable characteristics from a clinical perspective? Journal of health services research & policy. 2015;20(1 suppl): 26–34. 10.1177/1355819614557299 25472987

[6] Vahidi RG, Tabrizi JS, Iezadi S, Gholipour K, Mojahed F, Rasi V. Organizational Facilitators and Barriers to Implementing Effective Clinical Audit: Systematic Review. Journal of Pakistan Medical Students. 2013;3(1).

[7] Sinuff T, Muscedere J, Rozmovitz L, Dale CM, Scales DC. A qualitative study of the variable effects of audit and feedback in the ICU. BMJ quality & safety. 2015: bmjqs-2015-003978. 10.1136/bmjqs-2015-003978 25918432

[8] Christina V, Baldwin K, Biron A, Emed J, Lepage K. Factors influencing the effectiveness of audit and feedback: nurses’ perceptions. Journal of Nursing Management. 2016;24(0): 1080–1087. 27306646 10.1111/jonm.12409

[9] Grol R, Wensing M. What drives change? Barriers to and incentives for achieving evidence-based practice. Medical Journal of Australia. 2004;180(6): S57. 15012583 10.5694/j.1326-5377.2004.tb05948.x

[10] Gould NJ, Lorencatto F, Stanworth SJ, Michie S, Prior ME, Glidewell L, et al Application of theory to enhance audit and feedback interventions to increase the uptake of evidence-based transfusion practice: an intervention development protocol. Implementation Science. 2014;9(1): 1. 25070404 10.1186/s13012-014-0092-1 PMC4243714

[11] Hartley S, Foy R, Walwyn RE, Cicero R, Farrin AJ, Francis JJ, et al The evaluation of enhanced feedback interventions to reduce unnecessary blood transfusions (AFFINITIE): protocol for two linked cluster randomised factorial controlled trials. Implementation Science. 2017;12(1): 84 10.1186/s13012-017-0614-8 28673310 PMC5496161

[12] Baxter P, Jack S. Qualitative case study methodology: Study design and implementation for novice researchers. The Qualitative Report. 2008;13(4): 544–59.

[13] Michie S, Johnston M, Abraham C, Lawton R, Parker D, Walker A. Making psychological theory useful for implementing evidence based practice: a consensus approach. Quality and safety in health care. 2005;14(1): 26–33. 10.1136/qshc.2004.011155 15692000 PMC1743963

[14] Francis JJ, Stockton C, Eccles MP, Johnston M, Cuthbertson BH, Grimshaw JM, et al Evidence-based selection of theories for designing behaviour change interventions: Using methods based on theoretical construct domains to understand clinicians’ blood transfusion behaviour. British journal of health psychology. 2009;14(4): 625–46. 19159506 10.1348/135910708X397025

[15] Patey AM, Islam R, Francis JJ, Bryson GL, Grimshaw JM. Anesthesiologists’ and surgeons’ perceptions about routine pre-operative testing in low-risk patients: application of the Theoretical Domains Framework (TDF) to identify factors that influence physicians’ decisions to order pre-operative tests. Implementation Science. 2012;7(1): 1. 10.1186/1748-5908-7-52 PMC3522997 22682612

[16] Glidewell L, Boocock S, Pine K, Campbell R, Hackett J, Gill S, et al Using behavioural theories to optimise shared haemodialysis care: a qualitative intervention development study of patient and professional experience. Implementation Science. 2013;8(1): 118. 24098920 10.1186/1748-5908-8-118 PMC3851734

[17] Tuti T, Nzinga J, Njoroge M, Brown B, Peek N, English M, et al A systematic review of electronic audit and feedback: intervention effectiveness and use of behaviour change theory. Implementation Science. 2017;12(1): 61 10.1186/s13012-017-0590-z 28494799 PMC5427645

[18] Dyson J, Lawton R, Jackson C, Cheater F. Does the use of a theoretical approach tell us more about hand hygiene behaviour? The barriers and levers to hand hygiene. Journal of Infection Prevention. 2011;12(1): 17–24.

[19] Francis JJ, O’Connor D, Curran J. Theories of behaviour change synthesised into a set of theoretical groupings: introducing a thematic series on the theoretical domains framework. Implementation Science. 2012;7(1): 35. 22531601 10.1186/1748-5908-7-35 PMC3444902

[20] Francis JJ, Duncan EM, Prior ME, MacLennan GS, Dombrowski SU, Bellingan G, et al Selective decontamination of the digestive tract in critically ill patients in intensive care units: a mixed-methods feasibility study (the SuDDICU study). Health Technology Assessment. 2014;18(25). 10.3310/hta18250 PMC4967810 24775071

[21] Francis JJ, Duncan EM, Prior ME, MacLennan G, Marshall AP, Wells EC, et al Comparison of four methods for assessing the importance of attitudinal beliefs: an international Delphi study in intensive care settings. British Journal of Health Psychology. 2014;19(2): 274–91. 10.1111/bjhp.12066 24112280

[22] Graham-Rowe E, Lorencatto F, Lawrenson JG, Burr J, Grimshaw JM, Ivers NM, et al Barriers and enablers to diabetic retinopathy screening attendance: Protocol for a systematic review. Systematic Reviews. 2016;5(1): 134 10.1186/s13643-016-0309-2 27515938 PMC4981960

[23] Ivers NM, Grimshaw JM. Reducing research waste with implementation laboratories. The Lancet. 2016;388(10044): 547–8. 10.1016/S0140-6736(16)31256-9 27511773

