# Supplementary material for: How do hospitals respond to feedback about blood transfusion practice? A multiple case study investigation
Source: PLoS One. 2018 Nov 1;13(11):e0206676. doi: 10.1371/journal.pone.0206676 (PMC6211710; doi:10.1371/journal.pone.0206676)
Supplement: S1 Appendix — (DOCX) [file pone.0206676.s001.docx]

[PART 1: General transfusion practice/knowledge]

1. **Firstly can I begin by asking how long you have been working at [hospital]?**

*If less than 5 years – where did you work before this hospital and for how long?*

1. **And just briefly, what is your role in the blood transfusion process?**
2. **How much experience do you have in blood transfusion?**
3. **Do you personally make decisions to transfuse?**

*How often do you make these decisions?*

*If don’t make the decision, are you expecting to in the future?* *Who does make the decision? Do you have influence over other people’s decisions to transfuse?*

1. **Are there hospital policies or algorithms to inform transfusion decisions and practice?**

*So is there a document?*

*Is the policy discussed? Who with?*

*Do you know which bits of the policy are complied with?*

*Are patients ever involved in decisions to transfuse?*

*(If don’t have a policy), do you have any instructions or documents that you would go to?*

*[If junior doctor] during your induction onto this ward, did anyone talk to you about the hospital’s blood transfusion policy? Can you talk me through what was said?*

**I just want to clarify that for the purpose of this interview, when we refer to audit and feedback, we are referring to the National Comparative Audit of Blood Transfusion.**

1. **How many blood transfusion audit and feedback cycles can you recall being involved in?**

*If don’t know what National Comparative BT Audit is, show them laminated card*

1. **Do you know who analyses the audit data and prepares the feedback?**

*What do you think about the audit standards? Do you find them credible?*

*Do you remember any standards from a previous audit?*

*How credible do you think the feedback is?*

1. **What are your general opinions about the National Comparative Audit of Blood Transfusion process?**

*Did you find it useful?*

*Was it worth the effort?*

*In general, do you think audit and feedback might change your practice? In what ways?*

1. **Compared to other tasks that you have to do, where would you rank audit and feedback in terms of priority?**
2. **To what extent do you think your views are shared by the team**?
3. **Is there someone who is responsible for receiving the feedback materials and feeding these back to the team?**

*If yes - What is your understanding of their role in feeding back materials?*

1. **What influence do these individuals have over the group’s [***transfer term from answer to Question 9 above***] response to audit feedback?** *[ask directly if it is their role]*

*Perhaps down to each individual?*

PART 2: Feedback processes

**We’re now going to explore feedback of audited data in more detail. We will come back to some of the points you have raised later and discuss if there are ways in which the process could be improved.**

1. **Can you think about the most recent blood transfusion audit and feedback cycle that you were involved in. Can you talk me through the feedback process?** *(N.B: use any follow ups from below that are not covered in the answer, skip to Q27 if not* *previously involved in A&F)*

*How long ago was that?*

*At this hospital?*

*Were you aware when the feedback became available?*

*Did you have access to the feedback?*

*How easy was it to access the feedback?*

*And what happened when feedback became available?*

*Did you read the materials?*

*Do you remember which parts of the materials you looked at?*

*Who did the feedback come from?*

*What was the timeframe between an audit and receiving the feedback?*

*How valid do you think the audit data were at the time they reached you?*

1. **How [else] did you have access to feedback?**

*Did you receive feedback individually or as a team?*

*Who else had access to feedback?*

*Of the materials that you had access to, which stand out in your memory?*

*What were the main features of the materials that you saw?*

*[What mode was the feedback in? for example, verbal, electronic, presentation…]*

*Thinking back to the most recent blood transfusion audit and feedback cycle you were involved in, do you remember the recommendations? Were there parts of the recommendations that were less clear than others? If so, which?*

1. **Did you discuss the feedback materials with any of your colleagues in the hospital?**

*Who did you discuss feedback with?*

1. **Can you talk me through how feedback materials were discussed?**

*And did this occur at the team level?*

*Did you adapt the materials in any way? How? How much time does that take?*

*Did you create any additional materials from those that were available? What were they? How much time does that take?*

1. **Was there a specific meeting where you discussed the feedback?**

*Who attends this meeting?*

*How often do they occur?*

1. **And did you discuss performance in relation to other hospitals?**

*How important was discussing your performance in relation to other hospitals?*

1. **If feedback suggested that changes to practice or policy needed to be made, would you have influence over such changes?**

*[If yes] who would you be able to influence?*

*How would you go about making changes?*

*Which changes would be easy and which more difficult?*

*How receptive would your clinical colleagues be to making changes that you recommend?*

*If interviewing the TP/equivalent – In what ways are you supported in making changes following feedback? Who provides this support?*

*[if no] who is involved in making changes?*

*How would they go about making changes?*

*Which changes would be easy for them and which more difficult?*

1. **Were you aware of any ways in which change in practice was encouraged?**
2. **Did you set any goals in light of feedback?**

*What were they?*

*How did you decide what they were?*

*Were these individual or team? Any examples?*

*[if no goals, why did you decide not to]*

1. **Did you make any plans on how to change your practice or procedures to target these goals?**

*And can you remember those plans now?*

1. **And did you act upon those plans?**

*Could you remember those plans when you were managing a particular patient, in the rush and chaos of the daily hospital context? If so, did you manage to implement any? If so, which?*

*[if not in their job role], what about people within your team, do you think they could remember the plan? Do you think they acted upon the plan?*

1. **Did the action plan make a difference; if so how?**
2. **Did you keep monitoring? If so, how?**

1. **Did you feel you made progress towards your goal?**

*Can you talk me through that?*

1. **Do you think the team was confident in changing their blood transfusion practice in light of feedback?**
2. **Do you think the team had the skills to change their blood transfusion practice in light of feedback?**

*‘non-technical’ skills such as communication? Goals (achievable, measurable)? Action plans?*

1. **What do you think are the downsides of changing blood transfusion practice in light of feedback?**

*What about benefits?*

1. **Are patients ever involved in feedback?**
2. **Can you see a role for the patient perspective in relation to changing blood transfusion practice?**

*Can you elaborate on that?*

1. **If comparative data from an audit were made available to patients would this influence your policy or transfusion practice?**

*If yes, how?*

PART 3: Ways in which the process could be improved

Thank you for your input so far. In this final part of the interview we’re going to discuss if there are ways you think things could be improved, and how we might be able to help facilitate this.

We’ve spent a lot of time talking about what happened during your most recent audit and feedback cycle and how materials were accessed and discussed.

1. **In your opinion, how could the feedback process be managed better?**

*(prompt any of the below if not mentioned)*

*Receiving feedback*

*Reading feedback*

*Discussing feedback*

*Acting upon feedback*

**So thinking back to the purpose of this study, we are interested in how we might be able to enhance the process of audit and feedback**

1. **Are there any constraints to the feedback process that we would need to address or work around if we were to make changes? ( e.g. resources, time)**

*And do you have any suggestions for ways in which we could address or work around these?*

1. **We are also wondering if feedback from an audit could be discussed in any other meetings. Are there any key meetings where you discuss general practice with your colleagues?**

*Who attends?*

*How often do they occur?*

*FEEDBACK STRATEGIES – GO THROUGH SOME OPTIONS/IDEAS WITH PARTICIPANT AND GET THEIR OPINIONS. Have a laminated card as an example for each of these. Are any of these strategies currently used in your hospital?*

- *Jazzy document / plain paper document*
- *Action plan templates*
- *Changing forms for ordering blood products [do they order online?]*
- *Powerpoint presentation in transfusion meeting? Other meeting?*
- *Text message prompts (key recommendations)*
- *BT champion who keeps raising the recommendations in meetings*
- *QuickAudit*
- *Reminder Posters*
- *Other kinds of meetings*

*(If an engaged individual*) **– would you mind me coming back to you at a later stage with some materials aimed at making this an easier process and we could have a chat about if they would be useful?**

**If participant has to make an early exit to the interview, ask if they would be willing to answer a couple of additional questions by email (particularly around the potential feedback strategies)**
