# Supplementary material for: How do hospitals respond to feedback about blood transfusion practice? A multiple case study investigation
Source: PLoS One. 2018 Nov 1;13(11):e0206676. doi: 10.1371/journal.pone.0206676 (PMC6211710; doi:10.1371/journal.pone.0206676)
Supplement: S2 Appendix — (DOCX) [file pone.0206676.s002.docx]

**Meeting Type: Hospital Transfusion Committee** Start time:

Number of attendees: End time:

Job roles present (number of each):

Anyone noticeably missing:

|  |  | **General comments** |
| --- | --- | --- |
| Following a standard format? |  |  |
| Agenda? |  |  |
| Time for possible WS1b delivery? |  |  |
| Objectives of the meeting?  Clear?  Explicit? |  |  |
| **Functions of the meeting** |  |  |
| Behavioural regulation? |  |  |
| Social influence? |  |  |
| Documentation? |  |  |
| Case load/patient management? |  |  |
| Clinical decisions? |  |  |
| **Group processes** |  |  |
| Leadership style? |  |  |
| Persuasion? |  |  |
| Patient or safety focused? |  |  |
| One main speaker or discussion? |  |  |
| Generate ideas or just informed? |  |  |
| Seniority evident? Junior role? |  |  |
| How are decisions made? |  |  |
| Relationship between TP &… | Junior staff:  Senior staff: |  |
| **Body Language/non-verbal** |  |  |
| Chair/leader   - Facial expressions - Body language | Body language |  |
| Other audience members   - Facial expressions - Excited to be there? - Engaged? - Clock watching? |  |  |
| **Action points** | Total: |  |
| - Are they explicit? |  |  |
| - Responsibilities for whom doing what? |  |  |
| - Where do they look to for action points | Regional meeting?  Ward level?  Senior staff? |  |
| - Time limited? |  |  |
| - Any specific to A&F - Delegated to…? |  |  |

Sections of the meeting and their duration:
