# Supplementary material for: How do hospitals respond to feedback about blood transfusion practice? A multiple case study investigation
Source: PLoS One. 2018 Nov 1;13(11):e0206676. doi: 10.1371/journal.pone.0206676 (PMC6211710; doi:10.1371/journal.pone.0206676)
Supplement: S1 Table — (DOCX) [file pone.0206676.s003.docx]

| **Theme** | **Frequency of participants per case** | | **Case1** | **Case 2** | **Case 3** | **Case 4** |
| --- | --- | --- | --- | --- | --- | --- |
|  | Hospital Transfusion Committee (HTC) (total possible n=16) | Wider Hospital  (total possible n=9) | (R = researcher) |  |  |  |
| **Knowledge** | | | | | | |
| **Staff (do not) know about NCA audits** | Case 1: 3  Case 2: 3  Case 3: 5  Case 4: 4 | Case 1: 4  Case 2: 2  Case 3: 2  Case 4: 1 | “The only two we haven’t participated in is the Collection Audit that was here and the Medical Audit”  [R: do you know what the national comparative blood transfusion audit is?] “I wouldn’t really know a huge amount about it. I’m sure it’s got something to do with the amount of transfusions that take place” | “I am, Yes… we try to participate in all of them”  “I know it exists...That’s about as far as it goes, though… I don’t think I’ve ever seen the results of transfusion audit feedback” | [R: So how many blood transfusion audit and feedback cycles can you recall being involved in?] “There’s been a lot. More recently the anti-D national comparative audit. So, we’ve always been involved and always participated and sometimes actually been the pilot sites for the audit’s pre as well as the regional”  [R: any experience of NCA?] “I’m afraid not, no” | [R: how many National Comparative Audit cycles have you been involved in that you can recall?] “Practically I think the only one – well obviously there’s been some like the O-Negative one, I didn’t particularly do the lab stuff but most of the others I’ve done”  [R: if we’re thinking about National Comparative Audit of Blood Transfusion, how many blood transfusion audit and feedback cycles can you recall being involved in?] “None I don’t think, not here anyway, not off the top of my head.” |
| **Staff (do not) have knowledge of blood transfusion practice** | Case 1: 3  Case 2: 2  Case 3: 2  Case 4: 2 | Case 1: 4  Case 2: 2  Case 3: 2  Case 4: 1 | “it’s just endless because they go, yeah, we should be doing it, I can’t believe we’re not and then…for whatever reason nothing happens”  “The problem is getting people to understand the transfusion thresholds” | ““these are general wards where they happen to have to give blood transfusions  and they’ve got the knowledge to be able to do that”  “Even more difficult than that is getting people to understand about giving alternatives to blood products and I keep getting referrals from the surgical team, “We’ve seen this person in pre-assessment, they’re anaemic, they’re due for surgery in two weeks’ time, I want to give them three units of blood” and having that discussion around, “Well you should be giving them iron; they’re iron deficient, anaemic but it’s still fine to go ahead with the operation in that timescale”, is a much more difficult conversation” | “we have a good policy. We have good education. We have good knowledge of blood products. We have good knowledge of where they are, where they’re available, how to access them via blood track. And also knowledge of the massive haemorrhage pathway” | “I mean, we always are trying to practise evidence-based medicine, and that comes through in my departmental practice. We try and practise that within transfusion, and so by doing that we’re trying to bring about best practice” |
| **Social/Professional role & identity** | | | | | | |
| **It is clear who is responsible for audit and feedback** | Case 1: 3  Case 2: 4  Case 3: 5  Case 4: 3 | Case 1: 3  Case 2: 2  Case 3: 1  Case 4: 1 | “I’m in a position where it’s my role. My two roles in life is to improve transfusion practice and not transfuse if it’s not necessary. So that’s what I do all day. So if it means me going round and looking at one ward or, at a great detail and finding why on earth they’re not doing it then that’s what I’ll do.”  “What’s my understanding of their role [the Charge nurse identified by the participant]? Well, I think as, you know, as senior people they have, you know, they have a responsibility to pick up on like these things, like the Audits and giving feedback to the junior staff so that we can improve in the care.” | “Well I think as a Transfusion Practitioner people look at me 100 per cent to decide which audit we do because I am the one to do the audit, regardless of whether I am interested in the audit or not . So I think they are dependent on me saying ‘that audit is relevant’ or not , and how we cascade the audit and whether it is beneficial”  “and the SPoT nurse would then have to go and disseminate it, because she is the so called face of transfusion she would have to do that for the rest of the hospital I would have thought. So I think the two big link ups would be the SPoT and the Chair of the HTC” | “I mean I personally don’t get involved having to do much of it. The transfusion nurses get involved in it quite a bit.” | [R: so do you know if there’s someone who’s responsible for receiving the feedback materials and then feeding those back to the team?] “Yes there is, well certainly for the transfusion ones, there’s our transfusion lead, I think it’s [Doctor] and then somehow I think [Consultant Haematologist] has also been volunteered for us co-ordinating our departmental audits. That’s that, and I think they either stood mainly responsible but also [Pathology Director] the Pathology Director also has the role in making sure that all the results get feedback to us” |
| **Key individuals are (not) at meetings to discuss and disseminate feedback to their specialties** | Case 1: 3  Case 2: 3  Case 3: 3  Case 4: 4 | Case 1: 1  Case 3: 2 | “The HTT is myself and [TP] and the senior Bio-Medical Scientists. The HTC is the one that we had yesterday, so it’s consultants from the specialities. Not all specialties but they are all invited – haematology consultant, myself, head of the HTC. There should be someone from risk management there but they don’t always come. So that’s the big sort of Trust wide cross-speciality meeting.” | “but I think if people turn up to the Transfusion Team and Transfusion Committee meetings, then I wouldn’t have to go to so many different avenues to get that sort of response and giving them that feedback for them to feedback to me” | “And even presenting at an audit meeting you might only capture a third of the people who might need to hear about it.” | “And the key areas you want people turning up - acute medicine, Gastrointestinal bleeds, general surgery orthopaedic surgery, our colleagues in the emergency department, they are all very busy but there is no representation and that is the big problem you see. So the people that we really need to be discussing with, they are not there” |
| **Some clinical disciplines are more receptive to change in practice** | Case 1: 3  Case 4: 4 | Case 2: 1  Case 3: 1  Case 4: 1 | “But in the lab they’re used to change all of the time. So it’s kind of easier for the lab because they know it goes from one to another to another – it’s absolutely fine. You can’t do that for other people and there is a distinct difference between lab staff and those outside the Trust or other within the Trust outside the labs work so…” 7] | “And not wishing to be “them and us” about it, I think changes that impact on nursing staff seem to be much easier to push through than decisions, than changes that impact on doctor’s practice. Nurses are more receptive to change, I think, in general” | “I think you know, as doctors we’re welcome to change.” | “If we say, actually, in theatre we’re going to use cell salvage for all these cases now, you find that nursing staff are much more receptive to advice and change than doctors” |
| **Having specialist nurses or champions has raised the visibility and dissemination of feedback** | Case 2: 2  Case 3: 2  Case 4: 2 | Case 2: 2 | **Silence** | “Change in practice is encouraged by my training and me being very visible. And because they hadn’t had that in the past five years, they see me as that leader and making sure ... especially the shop floor staff, the front line staff see me as having, since I have been here, making this improvement” | “Well the big – in my lifetime – the big difference in blood transfusion was appointing the transfusion nurses. We had one of the first blood transfusion committees in the country back in the 80s and we used to meet and discuss things and say, you do this, you do that but not a lot happened. And then we managed to get funding for the first transfusion nurse and she, the whole thing stepped up a gear then because she was able, well she was the focus. So, people could feed things into her and as I say she went round to departmental meetings introducing herself and giving, you know, the information she had at the time – and that made a big difference” ” | [R: And so a blood transfusion champion who raises the recommendations – would that be helpful, or do you already have one?] “I mean, I’d say that we’ve got a few local ones. There’s the HTT, I think, are all blood transfusion champions. One of my colleagues in the anaesthetic department is very actively involved – he’s a former chair of the HTC and he’s still actively involved in transfusion. So we do have a few local champions.” |
| **Beliefs about consequences** | | | | | | |
| **Audit and feedback does (not) influence practice change** | Case 1: 3  Case 2: 4  Case 3: 5  Case 4: 4 | Case 1: 3  Case 2: 2  Case 3: 2 | “So I think audit is an important part of, you know, running and developing a service, because it really does help you to, sort of, you know, drive forward the changes that you want and also to make other people, or allows you to go to other people and say, “Look, this is what we’re doing wrong or right” or whatever and what you can do about it.” | “I think we do quite well in these audits actually so I think if we found for instance if the patients weren’t wearing their wristbands and we were out of kilter nationally, we would have to change practice for that. I know that is not the case but I think ... I mean it is not particularly useful for us if we are doing everything perfectly well, because we are not learning anything from that, but if we found we were lacking somewhere, obviously this is a good way to highlight it” | “Well, it just basically gives us assurance whether we do, what we should be doing and then at least you can improve on practice if you’re not reaching those standards that have been set”  “But does the actual audit process change practice is the crux of the whole thing and maybe there are some audits that have been repeated but at the same sort of levels of poor practice is shown again and again. So it might suggest that behaviour isn’t being changed.” | “Audit and feedback is vital, because it does give us information and knowledge to make sure we are continuing good practice or adapting our practice to deliver best practice” |
| **Feedback highlights that change is needed to enhance patient safety and outcomes** | Case 1: 1  Case 2: 1  Case 3: 4  Case 4: 3 | Case 1: 2  Case 2: 2  Case 3: 2  Case 4: 1 | “If we’re going to change something we’re changing it for a reason. We’re changing it for patient safety, for quality. You know if it’s not going to improve quality or patient safety why would you do it? Apart from finance. But then it must not be detrimental to patients, so… And the feedback is so important because if you don’t know that it is working, how do you know it’s going on. How do you know it’s a success? You know, you need to know. It’s the whole, you know, plan and act and…” | “Patient safety! I mean, even here as you say, you can become blasé, and just because you know the patient really well and you see them twice a week, it does, you know errors can still occur and you really, it just, it does highlight that you should pay attention and you can’t afford to cut corners, really, in relation to transfusion” | “Well, the benefits you’ve got to say as long as it enhances patients safety and care and the quality and also the aspects of staff safety as well, you know, you’re protecting them as well by the mechanisms and changes you’re putting in place.” | “Well, I mean, in that one, we set the goal that not giving anti-D to a woman at the right time was a ‘never’ event. I’m not sure that it is actually listed as a ‘never’ event in the trust – we do have ‘never’ events, but the consequences can be so severe to mother and future children that we have systems and policies and each time when they analyse the adverse events, there were reasons why they’d been missed, but there was also plenty of opportunities where it need not have been missed, and we insisted that – no, we strongly advised there should be a change in practice, and that it should be achievable, to make sure that those events didn’t happen again” |
| **Audit and feedback does (not) reduce costs** | Case 1: 1  Case 3: 2  Case 4: 3 | Case 2: 1  Case 3: 1  Case 4: 1 | “Well, benefits are apparent, aren’t they? Reducing blood usage, cost. We’ve seen all of that improve over the last few years on the committee. | [R: what are the benefits of audit and feedback?] “improved efficiency, efficiency of blood stocks, cost saving” | “Hopefully, if we can achieve change, then that will improve... better use of a precious resource.” | “I guess it’s balancing a sort of the cost thing, so trying to you know if there’s any feedback that comes through that can reduce usage of blood transfusion and rationalise people’s usage, that sort of feedback’s very positive.” |
| **Motivation & goals** | | | | | | |
| **Other demands take priority over responding to audit and feedback** | Case 1: 3  Case 2: 4  Case 3: 5  Case 4: 3 | Case 1: 3  Case 2: 2  Case 3: 2  Case 4: 1 | “I mean I audit probably, okay so I do Medicines and Healthcare Products Regulatory Agency audits and things like that, but I probably spend a couple of days a month doing audits. So, when you look at everything else that’s actually pretty high priority.”  “Well I know audit and feedback was very important but whether… but, um, mmm, in terms of my work whether or not the audit and the feedback takes priority over that, no, I don’t think.” | “I think out of ten, I would put it like a six or a seven in priority. Because as you can see we have spent a lot of time discussing the audits and looking at ways of how to improve practice, so yeah, I would give it a seven out of ten in priority”  “I would quite like to rank it pretty higher up but unfortunately because of my clinical workload it is pretty much in the last quartile, because I think the clinical work and the education etcetera probably takes precedence. I hate saying that but that is the truth” | “I suppose it’s fairly high up. I think for me patient safety is the key one. Education and training of staff, the getting things right and review of our policies and procedures and then audit” | [R: So compared to other tasks that you have to do, where would you rank audit and feedback in terms of priority?] “Audit and feedback would certainly be in my top ten, but the top ones would have to be organisation of the clinics, organising when results come through, organising patients being admitted for procedures and surgery, making sure they have got the right plans in place. So audit, I would probably put that at about number five in my top ten”  [R: Compared to other tasks that you have to do, where would you rank audit and feedback in terms of priority?] “To be honest it does end up fairly low down the list, purely from a time factor; by the time you've sort of got through your day to day jobs, we don’t really have any time set aside for audits so it’s you know, evenings and weekends.” |
| **Staff are (not) enthusiastic about audit and feedback** | Case 1: 3  Case 2: 2  Case 3: 4  Case 4: 4 | Case 1: 1  Case 2: 2  Case 3: 2 | “Often we just don’t get round to it...It’s possibly, it sounds dreadful, possibly lack of enthusiasm by the time we get it.” | “It depends how it’s presented and how signed up people are to actually doing something with this information” | [R: to what extent would you say your views on audit and feedback are shared by the wider team?] “I think some of them would think it’s a necessary evil, some of them live for it. I think it just depends, really, on the person” | [R: to what extent do you think your views are shared by the team?] “And again, sometimes it’s hard to engage other clinicians in that – so an example was the medical transfusion recordkeeping, and [TP], our transfusion practitioner, that’s very important, she was very engaged. But trying to find a physician to engage with that – we managed it, but it was more difficult” |
| **I experience ‘audit fatigue’** | Case 3: 3  Case 4: 3 | Case 2: 1  Case 3: 1 | **Silence** | “I think there’s a bit of ‘audit fatigue’ within the NHS generally. There’s a lot of audits that really shouldn’t even be happening, because they’re ... you know, it’s just not meaningful and then nobody does anything with that information anyway, so there is a general feeling of ‘Oh god, not another audit’” | “I think there’s a danger that there can be too many audits, so that people get a bit audit fatigued. Particularly as we’re expected to do local and regional audits as well as national audits.” | “I think one National Comparative Audit a year would be you know sometimes is you know, at the moment so far you know we had Anti-D last year, then we’ve had this patient consent one, now we’re doing one on the red cell the one week trace of, well then that’s you know you just think gosh, I haven’t even finished this consent and patient information one and now I’ve have had to do another one in the middle of it, so I actually think there’s too many” |
| **Standards are (not) up to date, appropriate, or credible** | Case 1: 1  Case 2: 2  Case 3: 5  Case 4: 3 | Case 1: 2  Case 2: 1 | “So, and I know they always go to proper documentation, so they always go into published guidelines, and so therefore they are vaguely credible but my concern is they’re not always as up to date as they could be” | “The audit standards are very appropriate, what they ask you, can have some improvement but nothing I can pinpoint at the minute” | “I think many they are, a lot of the audit standards we use are from British Committee from Standards and Haematology which are really consensus statements rather than evidence-based standards, is the way that I would see that, so while they would make perfect sense to me, I can see that some are perhaps a bit too rigid, maybe. If someone says, “You should transfuse at a platelet count of 20”, someone transfuses at a platelet count of 21, to me that’s not wrong, but they’ve broken the audit standard because it’s not 20” | “I think generally speaking the audit standards are in fact clear and they are appropriate and we would definitely concur with what has been put down. We haven’t come across any audits where we have felt the standards were inappropriate” |
| **Feedback is (not) clinically appropriate, valid or credible** | Case 1: 2  Case 2: 2  Case 3: 4  Case 4: 3 | Case 1: 3  Case 2: 1  Case 3: 2 | “Oh God the one I remember is the O Neg one. I nearly had a fit at that one. Gosh that really did rattle a few cages. Oh, I’d forgotten that. That was one where they were looking at usage of O negs and we, our wastage is tiny but we were criticised for using O negs on O pos patients so they didn’t get wasted. And I don’t see that as a wastage and I don’t see that as an inappropriate use. And we didn’t do very well on that, at all. The kind of feedback was you are using inappropriately. Wastage is inappropriate, using blood isn’t” | “I think the feedback is certainly useful to us, and I am sure it is absolutely credible. I don’t believe there is any problem with the validity of the data, but its usefulness is really for us to take that on board and use it in the best way possible for us” | “From the national comparative audit, well, I would imagine if it’s all been fed back it should be credible from, you know, the clinicians are actually doing a lot of the data analysis and oversees the project so I think it would be credible.” | [R: how credible do you think the feedback is] “I think it is spot on really. The stuff we see coming back would concur with what we believe as well” |
| **The rationale for audit is (not) made sufficiently clear to staff** | Case 2: 2  Case 3: 3 | Case 1: 1  Case 2: 1  Case 3: 1 | “So I think audit is . . . I think there’s a lot of unnecessary audit but I think if you, I mean I’ve come from a research background, so I think that if you do very clearly define what the question you’re asking is and you can see some sort of intervention at the end of it, the audit is, it can be a very, immensely helpful way to get what you want done, or the changes you want done, done. Because I think it’s very difficult for other people to argue with data that is very, you know it can be very compelling as well.” | “I think…if things come out of audit and those results are shared with the staff on the ward you know at ward level, then I think that as long as it’s explained to them the rationale why, most people are receptive. It’s when you get this dictat that comes and says we’re not doing that anymore, we’re doing this, without any explanation, I think that’s where you get the resistance, so as long as the feedback is there in the first instance, I think you can say it works well” | “It’s just I think audits are good but you’ve got to support it with education and training because, yes, staff that have been here for a few years know all this but you’ve got new starters and it’s getting the message across to them and the only way you do that is by training, supporting, educating. They wouldn’t know the background of an audit or why it’s come about, they know that there’s a policy there and it’s compliance with that.” | **Silence** |
| **Memory, attention & decision processes** | | | | | | |
| **I (do not) remember feedback materials** | Case 1: 3  Case 2: 3  Case 3: 4  Case 4: 4 | Case 1: 2  Case 2: 1  Case 3: 1 | [R: any materials stand out in your memory?] “None particularly.”  R: what happened when that feedback became available, do you remember?] “Mmm. What happened? I can’t remember.” | “I think we’ve had it back now, I can’t even remember” | “off the top of my head I have hundreds of audits so I can’t really recall numbers” | “I have an idea at the time when it’s fresh in your mind, you’ve done the audit, I can, I know what our results are going to be because I’ve actually done it but the longer time goes and obviously I can’t remember it all” |
| **I notice only information that is new, ‘leaps out’ as different or is clinically relevant to me** | Case 1: 3  Case 2: 4  Case 3: 5  Case 4: 3 | Case 1: 3  Case 2: 2  Case 3: 2 | “I look at the bits that are relevant to me clinically more I guess.” | “I think I kind of just looked at my bit really, and I know that it was highlighting the lack of wrist bands, so, yeah” | “If you’re head of nursing or chief nurse you want to know what the salient points are  so you can look at what you maybe need to take back to your staff to disseminate out” | “I read the results. I don’t need to particularly read how it was done and what the background is because I’ve done it” |
| **Staff (do not) remember recommendations or action plans** | Case 1: 3  Case 3: 5  Case 4: 3 | Case 1: 3  Case 2: 1  Case 3: 1 | [R: and can you remember those plans…] “We still try to remember, but, you know . . . it sometimes, sometimes it gets kind of forgotten and still sometimes does happen” | R: do you remember action plans?]“Yes, ‘cause they tend to follow similar themes, I have to say” | “you finally think you’ve got it sorted in one area and move on to the next, then you find you have to go back to that area again. You have to have constant awareness of the action plan, of the way forward, and reinforcement or…almost like validation of the need for the action plan, on a daily basis. Or a weekly basis, anyway” | [R: Do you remember those plans now?]: “Not really” |
| **Environmental context & resources** | | | | | | |
| **We require sufficient staff to conduct audits and/or respond to feedback** | Case 1: 3  Case 2: 3  Case 3: 4  Case 4: 4 | Case 1: 3  Case 2: 2  Case 3: 2  Case 4: 1 | “We haven’t taken part in all of the audits – most of them, but not all of them – because sometimes we just don’t have time. And the timescale you’re given - this will probably be about 12 questions down – the timescale you’re given often isn’t reasonable. Because we’re busy labs, we can’t stop and do this on this specific day at this specific time. | “if they’re going to be making changes that are going to impact on the workload of the ward staff for example or mean that we have to do more competency assessments or whatever it is, training even, then they have to almost work around what we are going to be able to release in terms of staff to do that.” | “There is a gap. It’s the timing and it’s also consideration of what the regional teams are doing as well if they’re asking you to do an audit. Because at the moment we’ve got a red cell survey that’s due in a couple of weeks, the consent audit goes over 12 weeks and a lot of the time it’s the transfusion practitioners that are asked to collect the data and in some trusts they only have one practitioner. So it is difficult . I think they need to look at the timing, the impact it’s going to have on people and just to see what is happening regionally as well. There may be similar audits.” | “If you did want to come and present the feedback, you would have to fit around our timetable, because we have a very diverse group within the HTC, at least, and trying to get us all together in one room is difficult enough for our own meetings.” |
| **Social Influences** | | | | | | |
| **Feedback is (not) shared and discussed with the relevant staff** | Case 1: 3  Case 2: 4  Case 3: 5  Case 4: 4 | Case 1: 4  Case 2: 2  Case 3: 2  Case 4: 1 | “Then we, it depends on what the particular audit is, but I will always feed it back to the next Hospital Transfusion Committee.”  “The problem we have is disseminating that through the hospital.” | “when I get the feedback I tend to say ‘here, this is yours, what would you like help from me, but also what would you do about it?’, you know ... that kind of thing, so I kind of delegate in that sense” | “the difficulty of reaching every single person in the Trust  because in a Trust this size, even in all the middle grade doctors we can’t even access them by email because they all have, they don’t have Trust email accounts. They tend to have their own doctors.org email accounts and there isn’t a sort of a global email address.” | [R: And how would you go about making changes?] “Well, I would identify who the target group were that made the decisions that needed to be changed, maybe. I would then, via email – and I’m more a face-to-face person as well, meet people, to discuss the audit and present. I would present the relevant data from the audit to them, and present it in a way that maybe showed them what was falling below the standard, and maybe suggest ways that we could improve things to come back and meet that standard” |
| **Feedback should come from someone whom staff know or respect, to influence change** | Case 1: 2  Case 2: 3  Case 3: 4  Case 4: 4 | Case 1: 3  Case 2: 1  Case 3: 1 | “one of the ways to do it is to have local champions, who you know, you kind of, you feed the data to them and then you get them to shout it out to everybody else, and so that’s quite a good way to do it, or you know, you feed it to them and then they send the emails on, so it’s not coming from some anonymous body, it’s coming from someone who they know, they’re more likely to open it and read it if it’s from someone they know rather than just automatically delete it.” | “unfortunately I wasn’t the one that presented that information and the analysis of the report, so they kind of got their back against the wall” | [R: Are there any ways in which you feel that you are supported to make changes following feedback, and if so who provides this support?] “Again, it’s got to be multi-disciplinary, because a clinician won’t take kindly being told to change practice by a biomedical scientist, but if that biomedical scientist has the support of his clinical haematologist or whatever, then on a clinician to clinician basis, again it’s horses for courses, you would try to influence where you can” | “Well yes because I’m the one who generally does it, I always say I’ve got a feel for this and yes, they just will accept what findings there are” |
| **I (do not) have influence over practice change** | Case 1: 3  Case 2: 4  Case 3: 5  Case 4: 4 | Case 1: 4  Case 2: 2  Case 3: 2 | “And I do, I suppose, ‘cause again we…certainly not on Intensive Therapy Unit but in…the acute medicine bit… we have a ward round where everyone comes together and discusses the patients…for an hour, every single patient on the ward, so if there was some inappropriate transfusion thing going on at that time, then I’d pick up on it and I’d say, “Was that the right thing to do, that’s against hospital policy” and it’s a fairly good forum to actually educate the juniors and also to point out that… there are guidelines that we should be following. And deviating…okay to deviate from them, but with good reason. Not, you know, personal whim.” | “[TP] does seek my opinions on a lot of things, I think because she knows that I do a lot of transfusion, so I’m a good kind of sounding board for if she does want to change something, or if she wanted to try something new” | “We can influence, you know, the other nursing staff on the ward as well as the out-patients because we do have quite a lot of nurse prescribing that is actually beginning now. So they can order the blood products and prescribe | “Well, we can influence most of the consultant staff. [TP] can obviously influence nursing staff, and laboratory staff if we need to” |
| **Comparing our performance against national performance is (not) useful for identifying areas for improvement** | Case 1: 3  Case 2: 4  Case 3: 5  Case 4: 4 | Case 1: 3  Case 2: 2  Case 3: 2 | “Because they are, a lot of the feedback we get is very much on a national picture. So you’re comparing, you know, all the participants, whether they be from a big teaching hospital or a local district general hospital or a private hospital and you’re just getting a percentage out. And, you know, if you’re, you cannot be compared on a national basis to someone like a private hospital who only does like two transfusions a week” | “I think it’s something that is quite a focus actually in our local area because there’s lots of hospitals around here, so you know our peer group are very close whereas I think some other organisations their peers are further away. We have [Hospital A] down the road, we’ve got [Hospital B], [Hospital C], [Hospital D], [Hospital E] which are all in a similar catchment area to us and we also share boundaries with a lot of them in terms of the patients that come here and go there, so we do look at their position as it were” | “I think you have to put it into perspective. I think it’s more important to track and trend your own performance, but as we work on a regional basis, we have enough hospitals of similar repertoire that we can actually compare performance with” | “benchmarking is really useful, but then sometimes data has to be protected so that you don’t really know who you’re benchmarking against, and comparing yourself with like-for-like trusts is more valuable than comparing yourself with a trust that’s got a totally different sort of clinical activity to you. So getting as much information out there, and I think most people – whether it’s good practice or not – I think everybody benchmarks to see how you are performing, and getting as much data that allows you to do that is good, while still protecting confidentiality, I guess |
| **I do (not) have support from my colleagues to make changes following feedback** | Case 2: 3  Case 3: 4  Case 4: 4 | Case 3: 2 | **Silence** | I think I am supported in making these feedbacks, when an incident occurred, and that sounds really bad but it is the truth and it is not just here, it is my previous job as well. I am supported in some of the areas, which I am happy about, but I think that things that I really really wish I can improve on, as you can see with the training and things, I have got to fight for it myself | [R: How easy is it to make changes in light of feedback]?“Easy would be to start with myself. But I think everybody would be actually resisting change to a varying extent.” | [R: So to what extent do you think your views of audit and feedback are shared by your team?] “Well yes because I’m the one who generally does it, I always say I’ve got a feel for this and yes, they just will accept what findings there are” |
| **Staff use inductions, training sessions and study days to influence practice** | Case 1: 2  Case 2: 1  Case 3: 4  Case 4: 2 | Case 2: 2  Case 3: 2 | “I’m always looking at new, and I don’t think it’s just new meetings. I think what, you know, is looking at, there’s different sort of study days, I mean like, as I say, this morning I’ve just been in, so the first time, I’m teaching on the Trauma Intimate Life Study Day. So I think sometimes when it’s actually a formal Study Day and you can take some stuff it may be a bit more meaningful than just a meeting and sit there asleep or whatever” | “the results of the audits we would actually incorporate in the training  and we would actually give the staff and the nurses or doctors, whoever it was, the results of the audit, to show them where things had gone wrong and what we needed to do, so yeah, you used it as part of training as well” | “the other area we target anyway, we’ll sometimes put a power point in the mandatory training, you know, highlighting the feedback of audit. And that impacts on a wider range of staff” | “I think the other way forward would be, the best place to start is with the Junior Doctors, is to be able to get more teaching sessions with the Junior Doctors, but that is difficult because there is so much training that goes on, so much mandatory training covering all aspects of hospital care, it’s getting access to them, but I think that is the way ahead, because if you got access to the Doctors when they are F1’s, you can help them to understand why you are going down that avenue with this as the best practice and you can shape them for the future.” |
| **External sources, such as patients, regional meetings and authorities influence response to audit and feedback** | Case 2: 2  Case 3: 5  Case 4: 3 | Case 2: 2  Case 3: 2 | **Silence** | “I think one of the advantages of transfusion practice is because it’s quite kind of regulated, people tend to see if something’s coming from NHS BT, they kind of think, “Well, we don’t have a choice, we have to implement this”, so yeah, it helps if something carries national weight” | “That’s very much internally decided, perhaps with some influence from outside from the NHS BT, patient blood management team or the customer services team, by saying, you know, “These should be, perhaps, your key performance indicators. Perhaps you need ... ” | [R: And so a blood transfusion champion who raises the recommendations – would that be helpful, or do you already have one?] “An external one – coming? I’m not sure – sometimes people work on that kind of support, sometimes people are resentful of outsiders trying to come and tell them what to do. So probably a local champion is better than external one” |
| **Behavioural Regulation** | | | | | | |
| **We have to amend the feedback to make it relevant to our hospital** | Case 1: 3  Case 2: 3  Case 3: 5  Case 4: 4 | Case 1: 2  Case 2: 1  Case 3: 1 | “And therefore then you could target and say, okay, this is the National Audit, this is our Trust Audit and this is where you are within your particular sort of specialty. But the national feedback wouldn’t give you that detail, it would just say you as a Trust, so again it, that’s really where what you’re wanting to know is okay, I know that my Trust is 67% for 15 minute observations, but what is Surgery? What is Medicine? What is Emergency Department? And so that is what you need to do, I think, at local level to actually find out where things are going wrong.” | “Obviously every Trust may have a slightly different policy in this and I think there was a bit of an assumption that it was maybe a more a uniform approach, but I don’t think that necessarily is the case. I didn’t think all the questions and standards really ... didn’t really fit that well for the way we deal with things” | “Yeah, I think there’s a risk that the recommendations can be quite broad-based and not specific for your own hospital, which is why we try and translate the recommendations that are put in the national audit into more locally do-able actions. Some of the recommendations are aimed at a national level anyway to change, maybe, an educational learning package or write a, or prepare a poster to change practice or something. But then there are others that need to be done more locally. So we would adapt the national recommendations for local use” | “I think it’s good, but it’s always difficult, because the feedback comes back as a national feedback – it’s then applying that national feedback, and you get the unit level data as well, and it’s trying to decipher what it means for us – how good we’ve been at meeting the standard, how we benchmark against other hospitals.  So I think it’s quite good, but getting down to the nitty gritty of it takes a bit of work sometimes” |
| **We try to monitor practice by re-auditing, re-feeding back and following up** | Case 1: 3  Case 2: 4  Case 3: 5  Case 4: 4 | Case 1: 3  Case 2: 1  Case 3: 2 | “Well the ones with the stats, I gather stats monthly, I’ll go back and I’ll look at it. And then feed that back to the staff. The stats often go up on the board. And if they are not achieving what I think they should do either I’ll go back to them or I’ll send [TP] back to them – because she’s very good at nagging!” | “so if we implement and embed some change in practice, then we will do another three months and six months, then a yearly review so even if in the audit when there is recommendations and we have sat and made plans to implement them, if we say six months, then I will say we re-audit six months later or just do a survey six months later, or a spot check six months later to see if it is actually working or not, or if there is any improvements, or if it just needs to stay that way.” | “Well, we put all the actions onto our database and then we can pull off reports for clinical effectiveness meetings to show them how many outstanding actions for each directorate or each division and we have a trust clinical audit committee who oversees throughout the trust all divisions and all the outstanding audits and looks at the reasons. We have divisional leads as well that are supposed to oversee the audits.  So we actually monitor the action plans from our side because we know the timescale that they should be done and then we can actually ask them by email, “Have you done this?” and just an email back to say, “Yes, we’ve done it” for us is the evidence that we then put in our database” | [R: do you keep monitoring or would they keep monitoring?] “Yes,…I can’t think of exact examples, but you would, if you’re going to bring about change, you have to re-audit it, to see your change has had an effect, and it may be that there would be another National Comparative Audit, because in the transfusion within medicine, we did. So we had Audit 1 and then there was Audit 2, and I can’t remember whether it was 18 months, two years later. But for example…I don’t know if there will be an anti-D 2 if there’s not, we would be asking for a local one to be done, to see whether we’ve met the standard that we’ve said that should be met. So sometimes, you’d wait for NCA or sometimes you would just do a local one” |
| **We analyse our data and feed back or act immediately rather than wait for national feedback** | Case 1: 2  Case 3: 1  Case 4: 3 |  | “We don’t wait for them to come out because they take so long and by the time they come out they really, unfortunately, don’t apply anymore because it’s taken so long to do them. So what we do is, if there’s a National Comparative Audit, we do – either [TP] or myself depending on what the audit is and whether it’s lab based or hospital based – is we do the audit, we collect our own data at the time, number crunch that and report it. So we have results in a month whereas we might wait 18 months/2 years. So we know…what’s going on.” | **Silence** | “we’ve already done a lot of work in between within the trust working with the teams before the audit was planned so it’s just that information is way back and you’re working on actions that are maybe no longer applicable . You may have already done them” | “I mean sometimes we act before we get the feedback, so the one that we’ve just done on the use of Anti-D Immunoglobulin highlighted some internal problems you know with midwifery, that we detected at the data collection stage, so that’s had an impact already.” |
| **We (do not) set goals or make action plans as a team** | Case 1: 3  Case 2: 4  Case 3: 5  Case 4: 4 | Case 1: 3  Case 2: 2  Case 3: 1 | “once I’ve got the feedback then we, the Audit Department here want us to come together with an Action Plan to say, okay, what Standards did you not meet and what are you aiming to do about it? And so therefore you set time frames of when you want to review that, so that’s what we’ve done with the Anti D one, say, right…we want this amended, by this date we want to do this. So that’s what we’ve tried to do.” | “I think if the feedback is from an audit or something, then I think you have to use the information that you’ve gathered otherwise as I said there’s no point in doing the audit in the first place and even if that’s just going to be that you’re going to set a target for a reduction in something or an increase in something depending on what it is, then yes I think that’s something that we definitely do” | “Yes, I think so. I think so. For example, say the audit feedback was that 20% of our patients were not wearing a wristband, next time we audit this we need to get that down to less than 5% or whatever else, so yeah, I think there are. I think it’s difficult to say, “No, we’re not going to do this any more, but we need to improve by so much, at least”.” | R: So who decides what these {goals} are?] “Well depending on the recommendations and then we have goals, like I say I usually do the reports, take it to the HTT and HTC, you know what do we want to do? How are we going to do it? And then that’s what I fill in on the report form and who’s going to lead on it and the date” |
| **Support materials could be useful for some staff** | Case 1: 3  Case 2: 4  Case 3: 5  Case 4: 4 | Case 1: 3  Case 2: 2  Case 3: 2  Case 4: 1 | [R: like an App, so I think they developed an App in relation to one of their audits, but whether that would be useful for something?] “Well, I mean, it’s very trendy isn’t it, and, people, yeah, I think that wouldn’t be a bad idea. Yeah . . . It says engaging people. You can have the App but you need to get them to download and use it. So, yeah, but yeah, I could see that certain individuals would find that quite good.” | “A red cells app, I would almost certainly use it” | [Apps] “So these will be definitely useful because I see increasingly, I may still be more resistant to using the technology and the foundation year doctors are coming in with the iPhone to the bedside. So when I was training as a doctor, they would say if you would refer that those you don’t know. And then, so you should know it when you’re actually doing it. But right now they will be having their iPads out when they’re actually seeing the patient” | [R: QuickAudit tool for snapshot audit] “Yeah, that would certainly - I think that would be very useful because it saves us a lot of time having to come up with proformas in the first place, if there’s something that we can quickly fill in, you know, get a list of our patients, however, many patients, put the data in quickly and it’s all summarised or sort of analysed through the system. It would be very helpful. Save us poor registrars a lot of time!” |
| **We need or use strategies to remind staff of actions and recommendations** | Case 1: 2  Case 2: 3  Case 3: 4  Case 4: 3 | Case 1: 4  Case 2: 1  Case 3: 2  Case 4: 1 | “I mean, yeah, we have, I mean, as I say, the 15 minute observation thing, we’ve looked at other things that we can actually try and do to remind people to go back within 15 minutes. You know, things like setting a pump or little timer or something like that. Or putting something in the bag, units of blood, that when they go out, reminding them. So we’ve looked at various sorts of things.” | “biggest problem that we have with this single and double platelets,...I think ITU was the major culprit because in a single breath they would pick up the phone and say ‘can we have two pools of platelets’ ...we have been trying to discourage that for a while but I think if you put up stickers and posters and whatever in the ITU because we discovered that ITU was the major culprit asking for lots and lots of platelets” | “What they’re trying to develop in [region] is a platelet count coming into the requestant saying, “By the way did you know your last, platelet count was 25, last time, are you sure you want to order the platelets?” And then they have to over-ride it if they say, “yes” and then you’ve got an audit trail to show someone’s not following the guidelines which you can follow up” | “And on the request form there is, if I just show you, well there’s a label stuck on the back of this one but you can see all the indication codes for transfusion are there, the acceptable ones and they have to give us a reason here.” |
| **The time between data collection and feedback is (not) too long** | Case 1: 3  Case 2: 2  Case 3: 5  Case 4: 4 | Case 2: 1  Case 3: 1 | “One of my complaints about the national audits is that we don’t get feedback. We took part in a national audit on use of blood in liver disease and we still haven’t had the feedback from that yet. And there’s been -- and they’ve already started the next audit and we still haven’t had the results back from the last one which we find not very constructive.” | “we will do an audit and it then takes about six, seven, eight months to bring to the local platform  and then people have forgotten that we did the audit” | “the disconnect, between when you make the effort and when you get the results back. And that by that time you may already be onto the next audit and then don’t have time to implement the recommendations from the previous audit” | “how relevant it is when it comes out like, The Part 1, Part 2, when it’s six, nine months, I think it could probably – it loses its importance as in because you’re maybe talking about something that – you know we’re in 2012 and we did this in 2011 and maybe practice has changed anyway” |
| **Nature of the behaviours** | | | | | | |
| **Established practices make it difficult to implement change** | Case 1: 1  Case 2: 1  Case 3: 4  Case 4: 2 | Case 2: 1  Case 3: 1 | “I think it’s really difficult to change consultant practice. They’re entrenched and I think people, yeah, I genuinely think that, that it’s very difficult to change a senior doctor practise.” | I think one of the challenges is, it’s one of those things that people are very kind of set in a particular way, and so trying to get them to unlearn something that has been kind of drummed into them, it can be very, very difficult. | “You’re pushing against established practice, against, “We’ve always done it this way, why should I change?” you’re pushing against culture. Culture change is perhaps the hardest thing to actually change.” | “And habits you know – ‘I’ve always done it this way and it’s fine, so why should I change now? I’m 55 years old, I’ve only got five years to go.” |
| **The feedback materials are too long** | Case 1: 3  Case 2: 2  Case 3: 4  Case 4: 1 | Case 4: 1 | “I mean ideal is a front sheet that has all the information on it. So…an A4 of a synopsis of what happens and then…you can then choose whether to go on and read all about everything else. You can’t get rid of doing all of your methodology and what have you” | “I mean I think something like this [feedback report] is quite unwieldy…but as I said I think it needs to be what are the key points” | “Well I think concentrating the key messages in the executive summary, key, key, key points, and to me, although data was useful, it belongs in the appendix at the back” | “I mean I don’t know, every audit is an individual, but generally there’s too much. There seems to be a huge document to me and all I want to know is, what are our results because I know about the thing” |
| **The nature of transfusion behaviour itself can make it difficult to follow recommendations** | Case 1: 2  Case 2: 1  Case 3: 1  Case 4: 1 | Case 1: 3  Case 3: 2 | “If you book a patient in for a transfusion, then you get some sort of a pay-back for having them in hospital for that many hours. If you give them IV iron, they’re not in hospital long enough to be, to cover the cost of the iron. So, the longer you keep them, bizarrely, the more money you get for it.” | “I think those kinds of changes are difficult, not because they are particularly resistant but sometimes it is just technically difficult to bleed a two day old neonate, and give us all that blood. So, they have got some technical challenges that they have got to live up to, so I think that is probably a difficult one to go through” | “although it says that you check how effective your transfusion was, on the other hand many of our patients, if they are in in-patients that’s fine, they will be having some daily bloods. But if you’re having somebody who is an out-patient and you’ve given some blood and platelets, you’re not necessarily needing to bring them back the next day or, you know, asking them to wait to check an increment. Then it’s being rational and then pragmatic to say that, “Okay, I’m not checking it because it’s an out-patient”. So, I guess it is not possible in every one” | “In transfusion…it’s changing all the time, it’s never really static…it’s audit and then you get a new guideline out, yes so, it’s continual, striving to kind of meet the changes of practice that are required” |

Abbreviations: HTC – Hospital Transfusion Committee; HTT – Hospital Transfusion Team; NCA – National Comparative Audit; NHSBT – National Health Service Blood & Transplant; SPoT - Specialist Practitioner of Transfusion; TP –Transfusion Practitioner.
